# Supplementary material for: Personalised antimicrobial susceptibility testing with clinical prediction modelling informs appropriate antibiotic use
Source: Nat Commun. 2024 Nov 21;15:9924. doi: 10.1038/s41467-024-54192-3 (PMC11582675; doi:10.1038/s41467-024-54192-3)
Supplement: Supplementary file 1 — Supplementary information [file 41467_2024_54192_MOESM1_ESM.pdf]

Data cleaning step	Description
1	Standardisation of organism and drug terminology to that used by R ‘AMR: Antimicrobial Resistance Data Analysis’ package version 2.1.1 (available at https://cran.r-project.org/web/packages/AMR/index.html).
2	Removal of specimens with terms not recognised and parsed correctly by the R ‘AMR: Antimicrobial Resistance Data Analysis’ package.
3	Transposing of individual specimen results from rows with minimum inhibitory concentrations and interpretations S/I/R) to columns with S/I/R interpretations only, using the MIMER package version 1.0.1 (available at https://cran.rstudio.com/web/packages/MIMER/index.html).
4	Addition of organism grouping variables (e.g., genus, order) to facilitate subsequent data engineering.
5	Addition of intrinsic resistance patterns using the R ‘AMR: Antimicrobial Resistance Data Analysis’ eucast_rules() function.
6	Imputation of expected susceptible phenotypes based on available specimen susceptibility prevalence, using Bayes’ theorem with author-chosen prior for regularisation.
7	Filtering of dataset to only urine culture specimens with commonly uropathogenic organisms grown.
8	Removal of antimicrobial agent susceptibility columns with high proportions (more than five percent) missing data.
9	Bayesian simulation of antimicrobial susceptibility results with likely inferable reasons for missing data that were judged by the authors to be unlikely to introduce bias if imputed, using the technique described in step 6 (cefazolin for *Proteus mirabilis*, ciprofloxacin for *Enterococcus* species, piperacillin-tazobactam for common Enterobacterales).
10	Replacement of missing meropenem and trimethoprim-sulfamethoxazole results with a fourth ‘NT – not testable’ result class for *Enterococcus* species.
11	Deletion of rows with missing antimicrobial results that were missing in less than five percent of cases, and therefore likely to be missing completely at random.
12	The dataset was filtered to only the most recent AST result for each patient, to avoid cross-contamination of training, validation, and microsimulation study datasets with results from the same patients.
13	Microbiology specimens with multiple organism growth were collapsed to a single row, with results prevailing in the prioritisation order R > I > S > NT.
14	I results were reclassified as S, and NT results were reclassified as R.
15	The authors assessed all MIMIC-IV datasets for the presence of data that could plausibly be predictive of antimicrobial resistance (e.g., previous antimicrobial treatment exposure, previous detection of antimicrobial resistance – the table below displays the full list of variables included, grouped by their source datasets). Selected data was then engineered into either Boolean or standardised continuous feature variables.
16	Categorical predictor variables with more than two classes were transposed into separate Boolean dummy variables (e.g., a single ciprofloxacin susceptibility variable became three Boolean variables for the presence of susceptible [S], susceptible at increased exposure [I], or a resistant [R] result). AST outcome results of interest for the 13 antimicrobial agents remained as single variables with the two classes S and R.

Supplementary Table 1: The process used to clean the datasets used for the model development, microsimulation and sensitivity analyses. Numbers on the left represent stages in the sequential process.

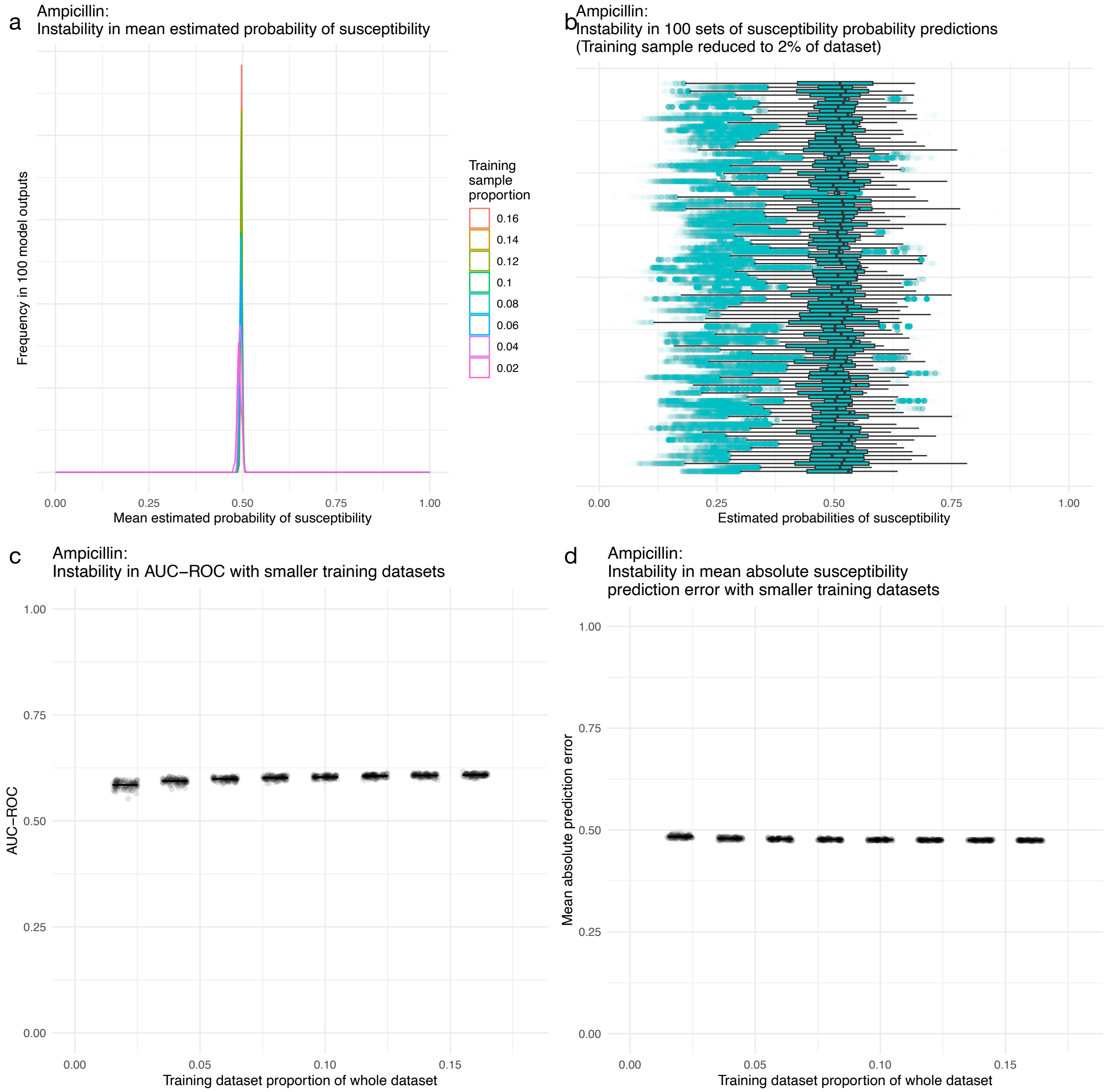

Supplementary Figure 1: Model stability analysis for ampicillin prediction model (n=23,812 patients)**.** Plot A displays instability in the mean estimated probability of susceptibility across the 100 model train-validate runs at a range of different training dataset sizes, with each dataset size represented by a different line colour and the height of the line representing the relative frequency of mean estimated probability in the 100 runs. Plot B displays instability in the distribution of estimated probabilities across the 100 runs, with each run represented by a blue box representing the interquartile range and median (central vertical line), black whiskers representing the full range excluding outliers (interquartile range multiplied by 1.5), and translucent blue points representing outliers. Plot C and D display instability in AUC-ROC values and mean absolute susceptibility prediction error respectively across the 100 runs for each training dataset size – grey points represent each train-test run with horizontal jitter applied for visualisation purposes, and horizontal black lines represent the arithmetic mean value across the 100 runs.

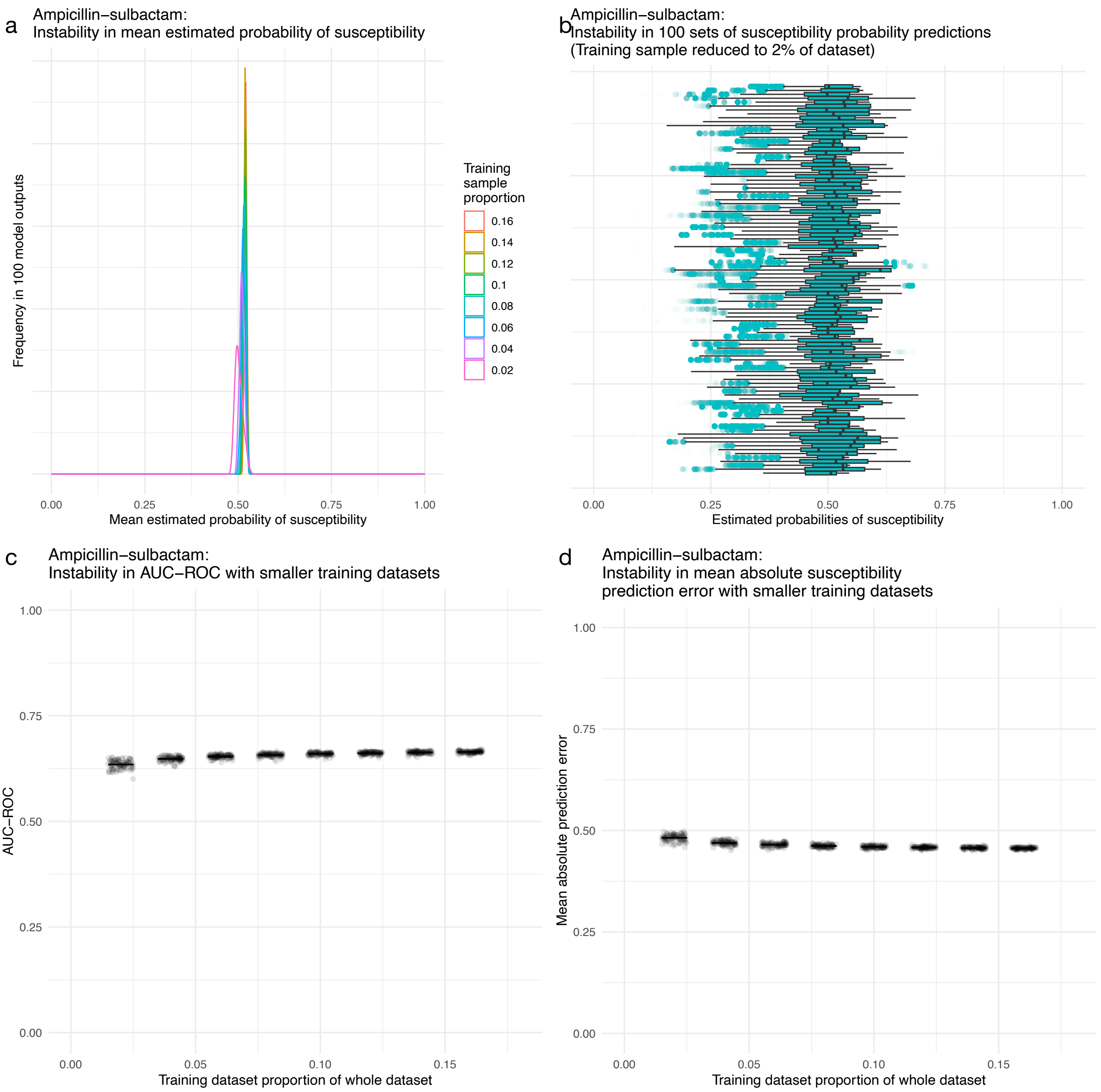

Supplementary Figure 2: Model stability analysis for ampicillin-sulbactam prediction model (n=23,812 patients)**.** Plot A displays instability in the mean estimated probability of susceptibility across the 100 model train-validate runs at a range of different training dataset sizes, with each dataset size represented by a different line colour and the height of the line representing the relative frequency of mean estimated probability in the 100 runs. Plot B displays instability in the distribution of estimated probabilities across the 100 runs, with each run represented by a blue box representing the interquartile range and median (central vertical line), black whiskers representing the full range excluding outliers (interquartile range multiplied by 1.5), and translucent blue points representing outliers. Plot C and D display instability in AUC-ROC values and mean absolute susceptibility prediction error respectively across the 100 runs for each training dataset size – grey points represent each train-test run with horizontal jitter applied for visualisation purposes, and horizontal black lines represent the arithmetic mean value across the 100 runs.

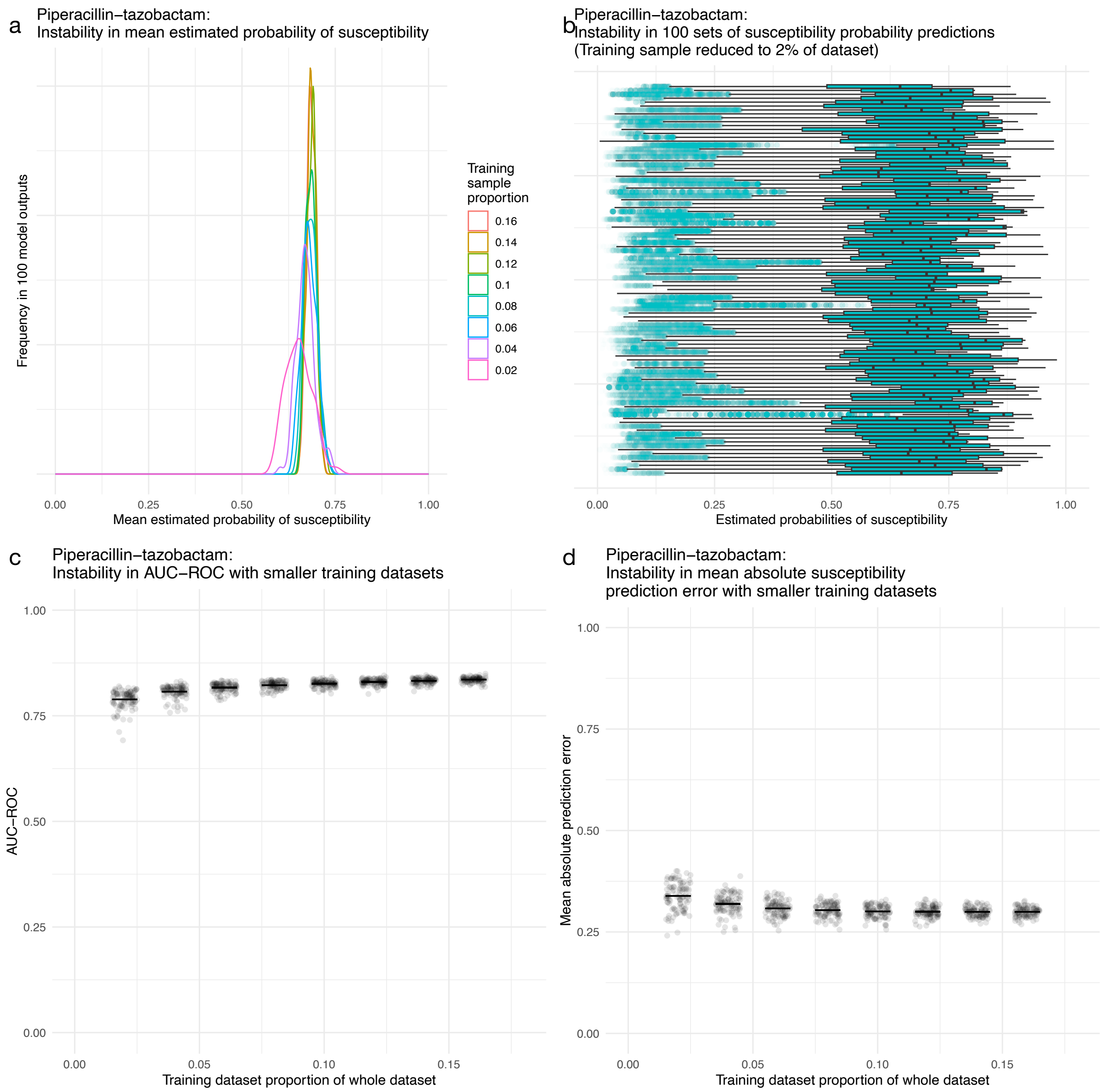

Supplementary Figure 3: Model stability analysis for piperacillin-tazobactam prediction model (n=23,812 patients)**.** Plot A displays instability in the mean estimated probability of susceptibility across the 100 model train-validate runs at a range of different training dataset sizes, with each dataset size represented by a different line colour and the height of the line representing the relative frequency of mean estimated probability in the 100 runs. Plot B displays instability in the distribution of estimated probabilities across the 100 runs, with each run represented by a blue box representing the interquartile range and median (central vertical line), black whiskers representing the full range excluding outliers (interquartile range multiplied by 1.5), and translucent blue points representing outliers. Plot C and D display instability in AUC-ROC values and mean absolute susceptibility prediction error respectively across the 100 runs for each training dataset size – grey points represent each train-test run with horizontal jitter applied for visualisation purposes, and horizontal black lines represent the arithmetic mean value across the 100 runs.

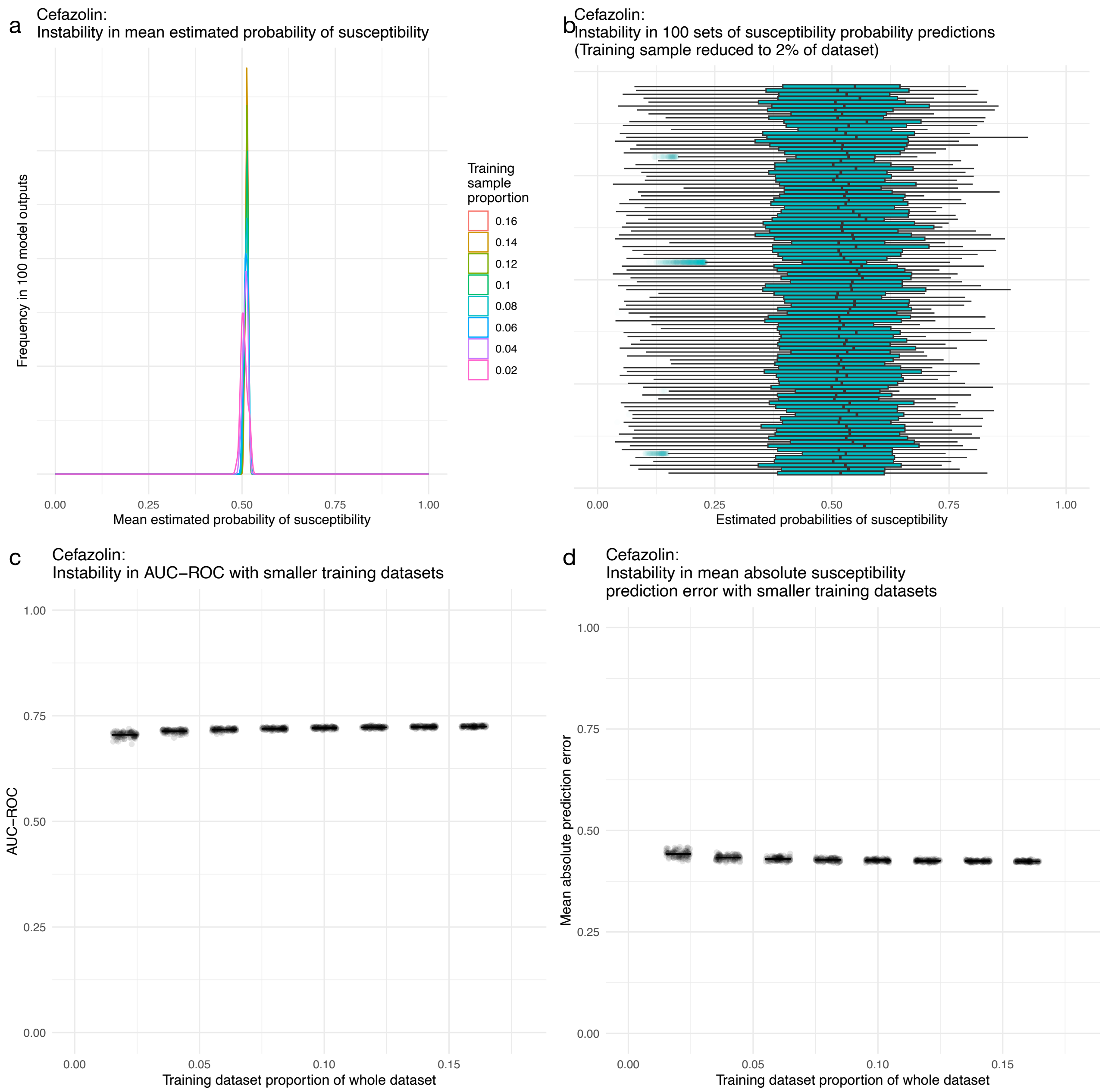

Supplementary Figure 4: Model stability analysis for cefazolin prediction model (n=23,812 patients)**.** Plot A displays instability in the mean estimated probability of susceptibility across the 100 model train-validate runs at a range of different training dataset sizes, with each dataset size represented by a different line colour and the height of the line representing the relative frequency of mean estimated probability in the 100 runs. Plot B displays instability in the distribution of estimated probabilities across the 100 runs, with each run represented by a blue box representing the interquartile range and median (central vertical line), black whiskers representing the full range excluding outliers (interquartile range multiplied by 1.5), and translucent blue points representing outliers. Plot C and D display instability in AUC-ROC values and mean absolute susceptibility prediction error respectively across the 100 runs for each training dataset size – grey points represent each train-test run with horizontal jitter applied for visualisation purposes, and horizontal black lines represent the arithmetic mean value across the 100 runs.

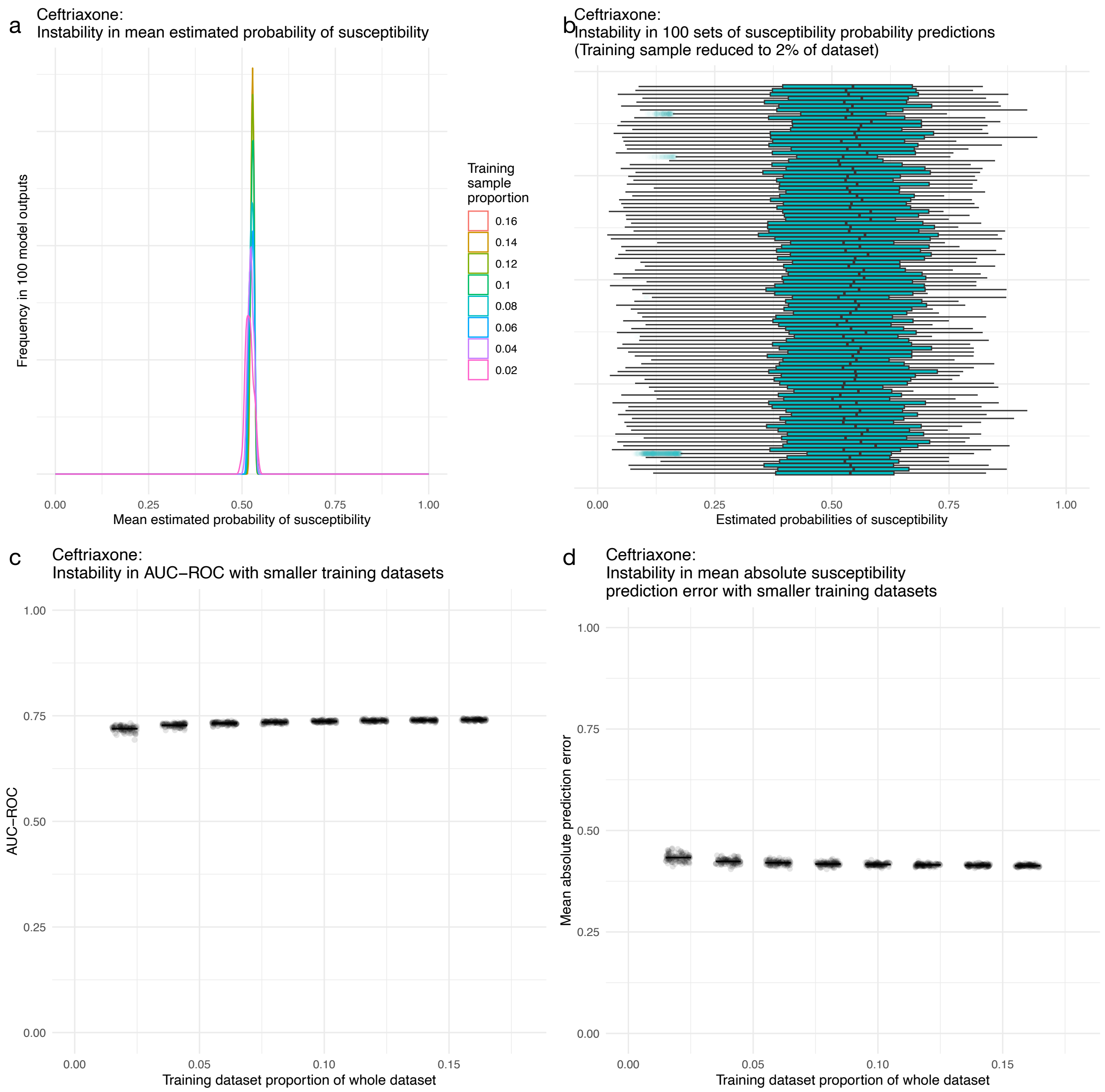

Supplementary Figure 5: Model stability analysis for ceftriaxone prediction model (n=23,812 patients)**.** Plot A displays instability in the mean estimated probability of susceptibility across the 100 model train-validate runs at a range of different training dataset sizes, with each dataset size represented by a different line colour and the height of the line representing the relative frequency of mean estimated probability in the 100 runs. Plot B displays instability in the distribution of estimated probabilities across the 100 runs, with each run represented by a blue box representing the interquartile range and median (central vertical line), black whiskers representing the full range excluding outliers (interquartile range multiplied by 1.5), and translucent blue points representing outliers. Plot C and D display instability in AUC-ROC values and mean absolute susceptibility prediction error respectively across the 100 runs for each training dataset size – grey points represent each train-test run with horizontal jitter applied for visualisation purposes, and horizontal black lines represent the arithmetic mean value across the 100 runs.

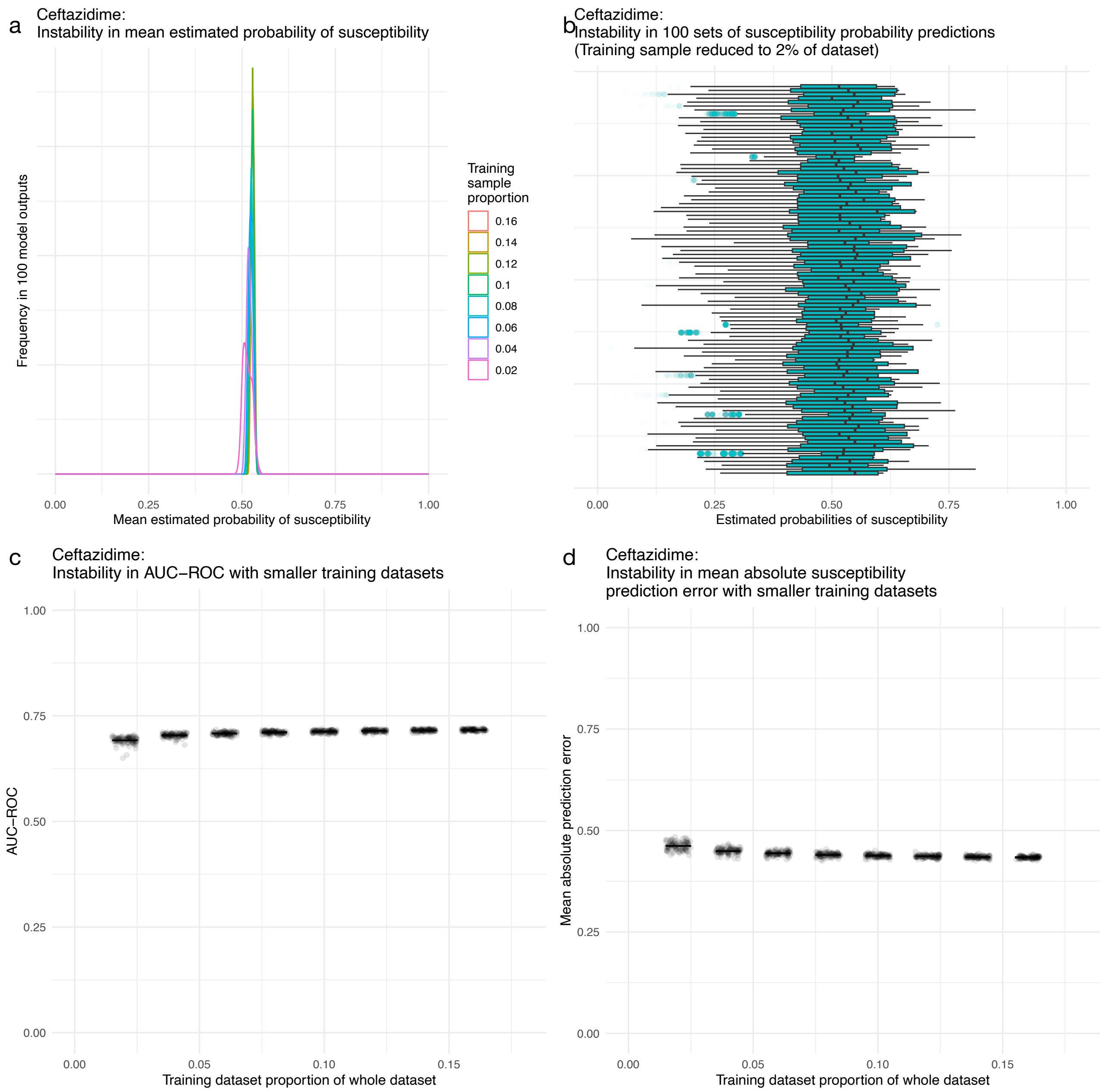

Supplementary Figure 6: Model stability analysis for ceftazidime prediction model (n=23,812 patients)**.** Plot A displays instability in the mean estimated probability of susceptibility across the 100 model train-validate runs at a range of different training dataset sizes, with each dataset size represented by a different line colour and the height of the line representing the relative frequency of mean estimated probability in the 100 runs. Plot B displays instability in the distribution of estimated probabilities across the 100 runs, with each run represented by a blue box representing the interquartile range and median (central vertical line), black whiskers representing the full range excluding outliers (interquartile range multiplied by 1.5), and translucent blue points representing outliers. Plot C and D display instability in AUC-ROC values and mean absolute susceptibility prediction error respectively across the 100 runs for each training dataset size – grey points represent each train-test run with horizontal jitter applied for visualisation purposes, and horizontal black lines represent the arithmetic mean value across the 100 runs.

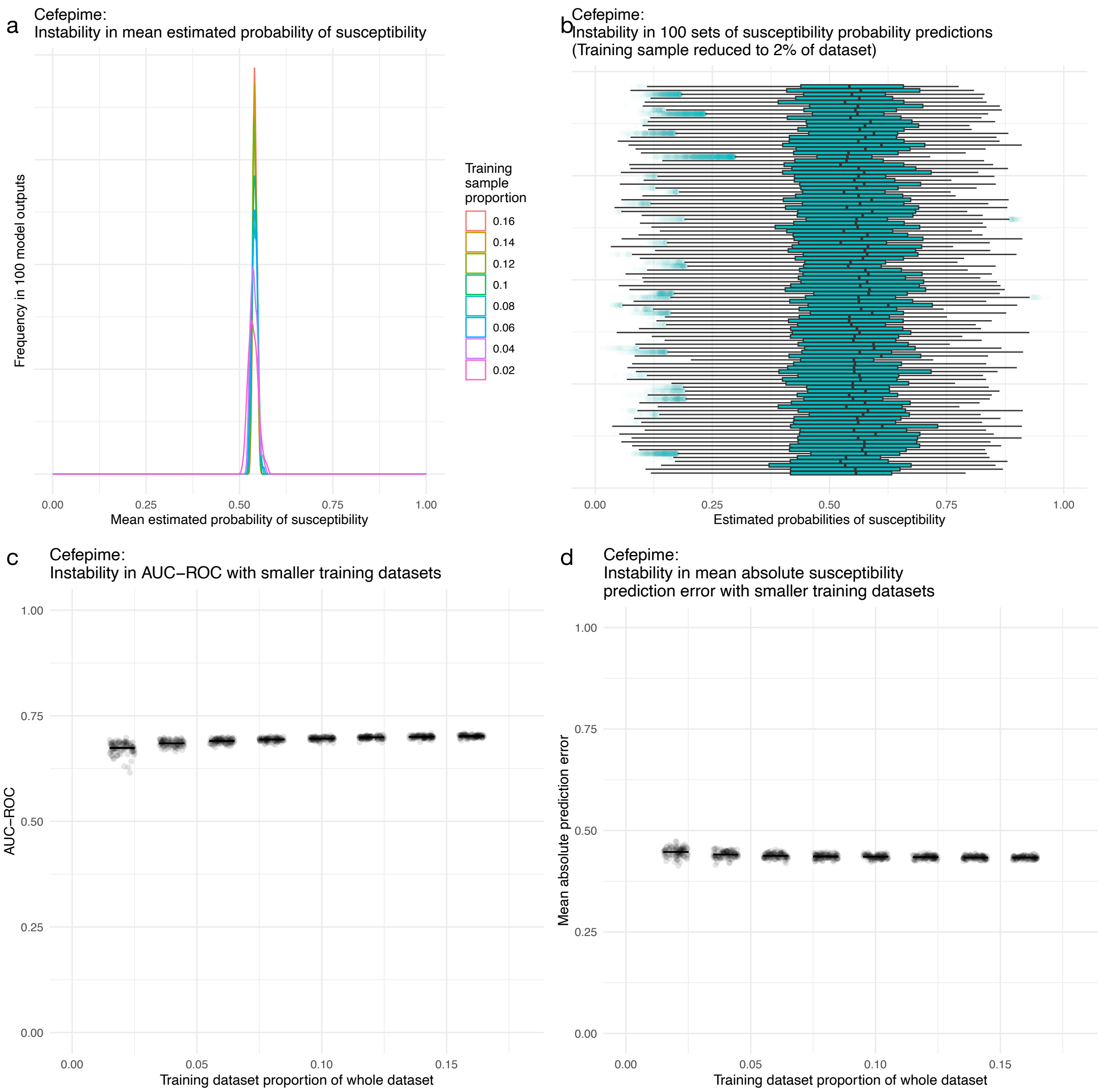

Supplementary Figure 7: Model stability analysis for cefepime prediction model (n=23,812 patients)**.** Plot A displays instability in the mean estimated probability of susceptibility across the 100 model train-validate runs at a range of different training dataset sizes, with each dataset size represented by a different line colour and the height of the line representing the relative frequency of mean estimated probability in the 100 runs. Plot B displays instability in the distribution of estimated probabilities across the 100 runs, with each run represented by a blue box representing the interquartile range and median (central vertical line), black whiskers representing the full range excluding outliers (interquartile range multiplied by 1.5), and translucent blue points representing outliers. Plot C and D display instability in AUC-ROC values and mean absolute susceptibility prediction error respectively across the 100 runs for each training dataset size – grey points represent each train-test run with horizontal jitter applied for visualisation purposes, and horizontal black lines represent the arithmetic mean value across the 100 runs.

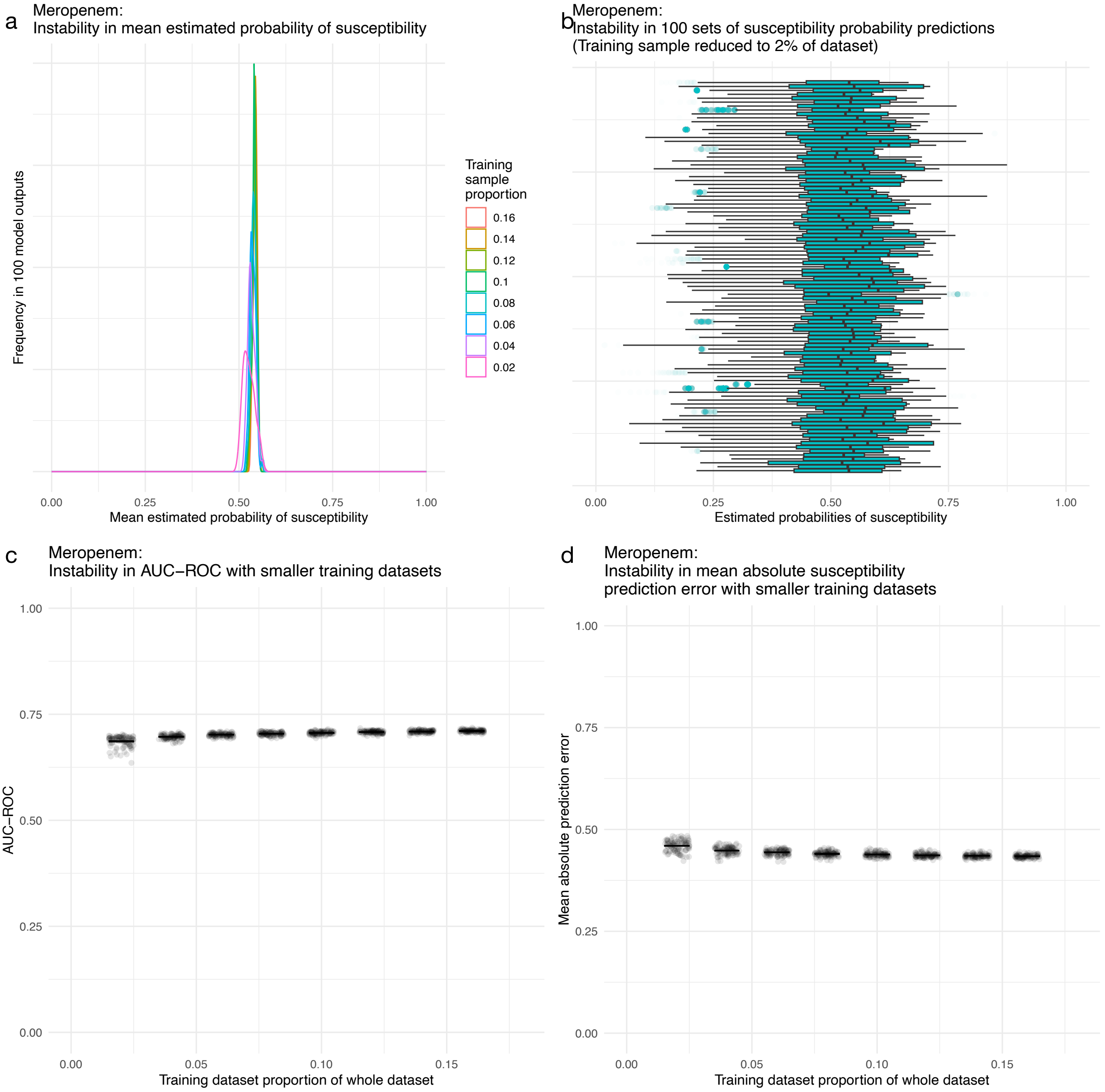

Supplementary Figure 8: Model stability analysis for meropenem prediction model (n=23,812 patients)**.** Plot A displays instability in the mean estimated probability of susceptibility across the 100 model train-validate runs at a range of different training dataset sizes, with each dataset size represented by a different line colour and the height of the line representing the relative frequency of mean estimated probability in the 100 runs. Plot B displays instability in the distribution of estimated probabilities across the 100 runs, with each run represented by a blue box representing the interquartile range and median (central vertical line), black whiskers representing the full range excluding outliers (interquartile range multiplied by 1.5), and translucent blue points representing outliers. Plot C and D display instability in AUC-ROC values and mean absolute susceptibility prediction error respectively across the 100 runs for each training dataset size – grey points represent each train-test run with horizontal jitter applied for visualisation purposes, and horizontal black lines represent the arithmetic mean value across the 100 runs.

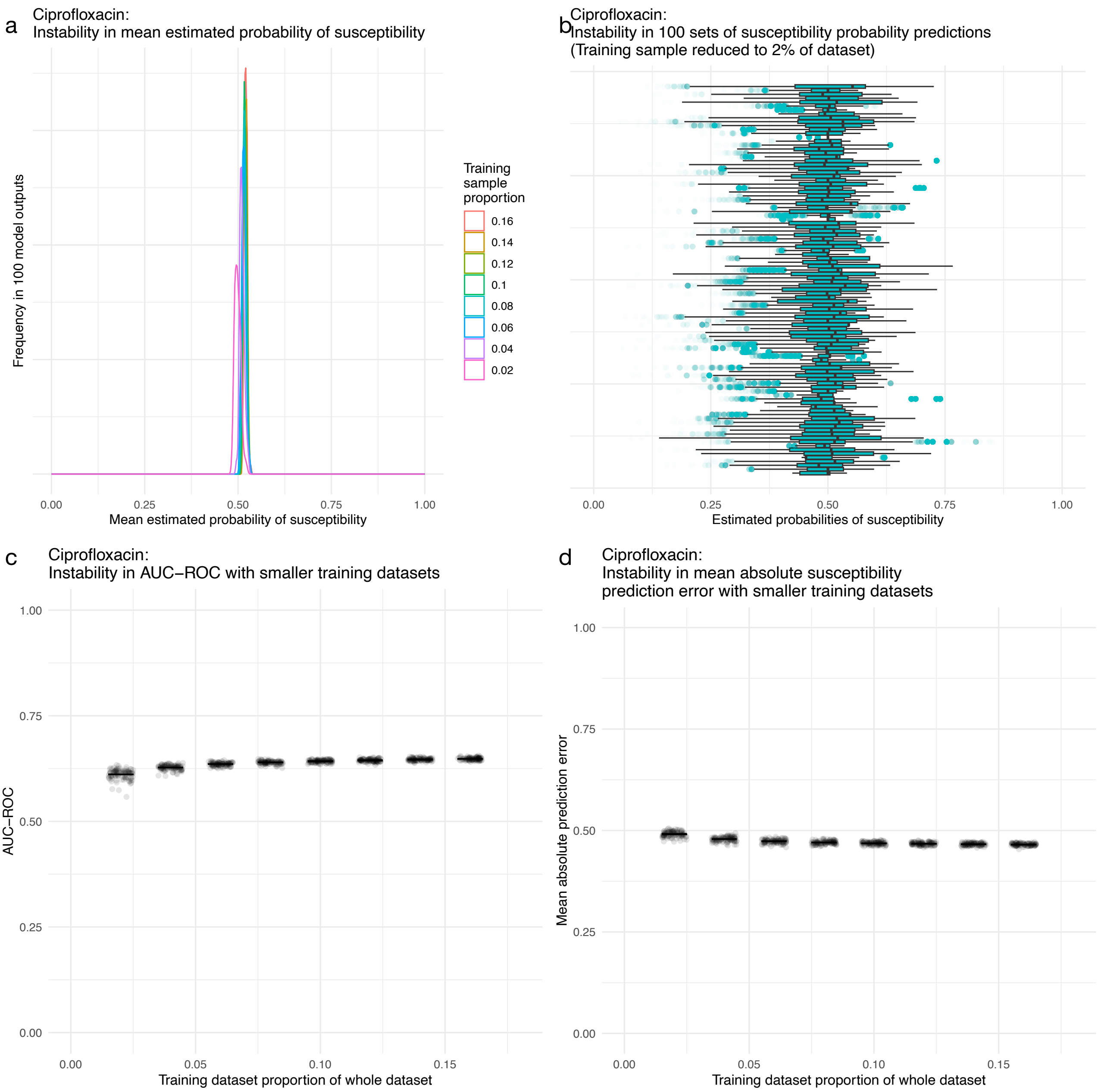

Supplementary Figure 9: Model stability analysis for ciprofloxacin prediction model (n=23,812 patients)**.** Plot A displays instability in the mean estimated probability of susceptibility across the 100 model train-validate runs at a range of different training dataset sizes, with each dataset size represented by a different line colour and the height of the line representing the relative frequency of mean estimated probability in the 100 runs. Plot B displays instability in the distribution of estimated probabilities across the 100 runs, with each run represented by a blue box representing the interquartile range and median (central vertical line), black whiskers representing the full range excluding outliers (interquartile range multiplied by 1.5), and translucent blue points representing outliers. Plot C and D display instability in AUC-ROC values and mean absolute susceptibility prediction error respectively across the 100 runs for each training dataset size – grey points represent each train-test run with horizontal jitter applied for visualisation purposes, and horizontal black lines represent the arithmetic mean value across the 100 runs.

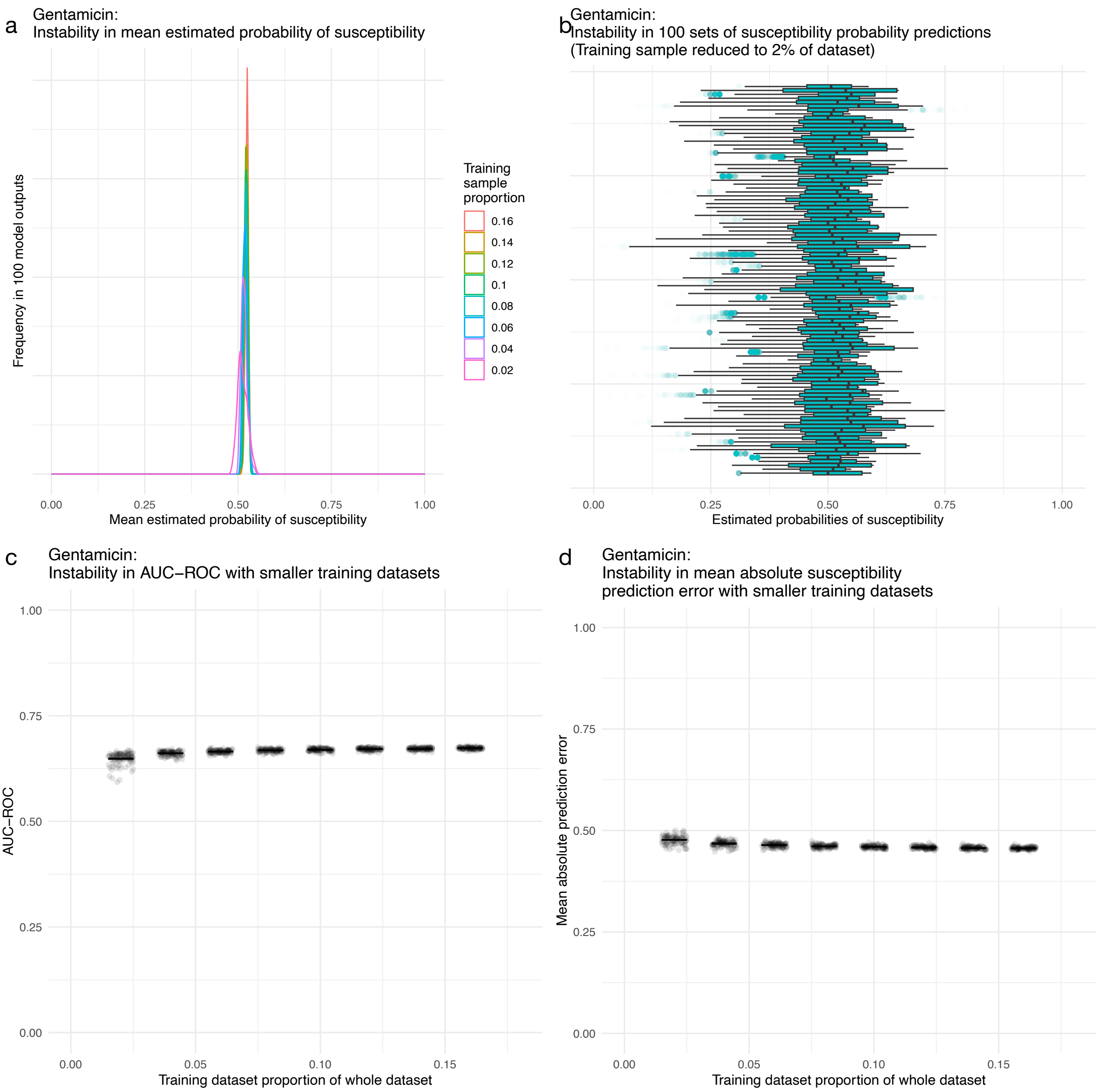

Supplementary Figure 10: Model stability analysis for gentamicin prediction model (n=23,812 patients)**.** Plot A displays instability in the mean estimated probability of susceptibility across the 100 model train-validate runs at a range of different training dataset sizes, with each dataset size represented by a different line colour and the height of the line representing the relative frequency of mean estimated probability in the 100 runs. Plot B displays instability in the distribution of estimated probabilities across the 100 runs, with each run represented by a blue box representing the interquartile range and median (central vertical line), black whiskers representing the full range excluding outliers (interquartile range multiplied by 1.5), and translucent blue points representing outliers. Plot C and D display instability in AUC-ROC values and mean absolute susceptibility prediction error respectively across the 100 runs for each training dataset size – grey points represent each train-test run with horizontal jitter applied for visualisation purposes, and horizontal black lines represent the arithmetic mean value across the 100 runs.

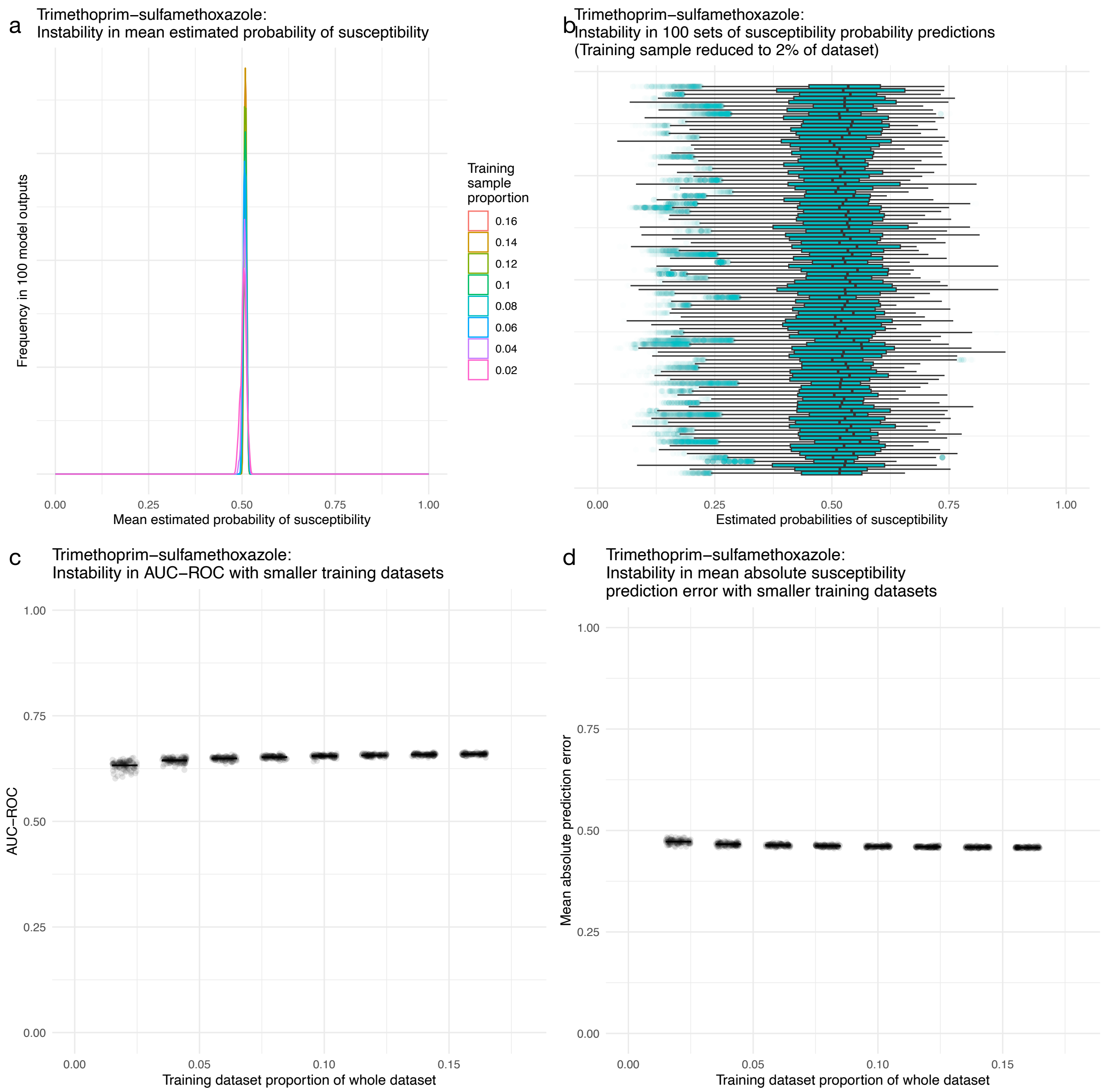

Supplementary Figure 11: Model stability analysis for trimethoprim-sulfamethoxazole prediction model (n=23,812 patients)**.** Plot A displays instability in the mean estimated probability of susceptibility across the 100 model train-validate runs at a range of different training dataset sizes, with each dataset size represented by a different line colour and the height of the line representing the relative frequency of mean estimated probability in the 100 runs. Plot B displays instability in the distribution of estimated probabilities across the 100 runs, with each run represented by a blue box representing the interquartile range and median (central vertical line), black whiskers representing the full range excluding outliers (interquartile range multiplied by 1.5), and translucent blue points representing outliers. Plot C and D display instability in AUC-ROC values and mean absolute susceptibility prediction error respectively across the 100 runs for each training dataset size – grey points represent each train-test run with horizontal jitter applied for visualisation purposes, and horizontal black lines represent the arithmetic mean value across the 100 runs.

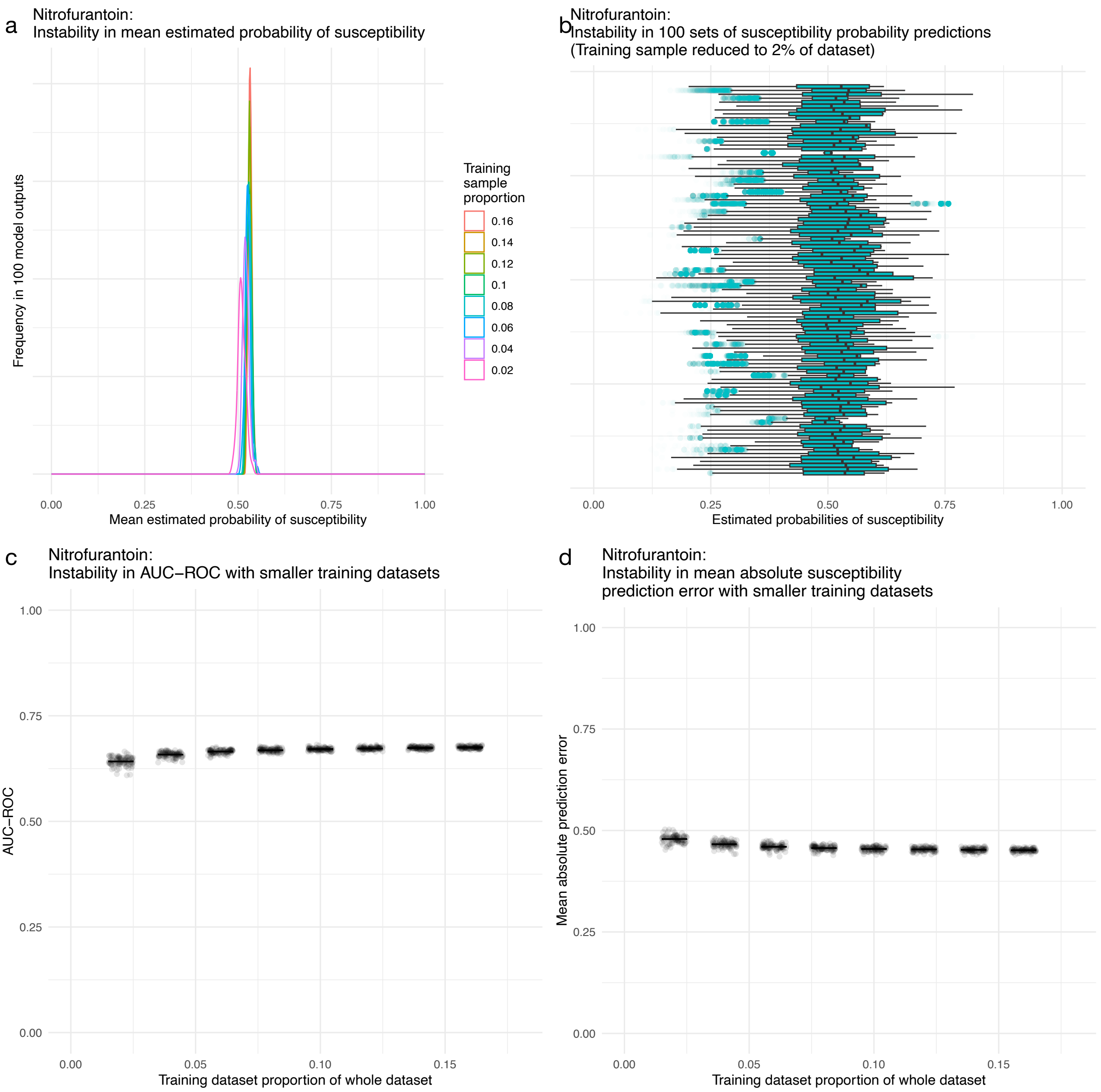

Supplementary Figure 12: Model stability analysis for nitrofurantoin prediction model (n=23,812 patients)**.** Plot A displays instability in the mean estimated probability of susceptibility across the 100 model train-validate runs at a range of different training dataset sizes, with each dataset size represented by a different line colour and the height of the line representing the relative frequency of mean estimated probability in the 100 runs. Plot B displays instability in the distribution of estimated probabilities across the 100 runs, with each run represented by a blue box representing the interquartile range and median (central vertical line), black whiskers representing the full range excluding outliers (interquartile range multiplied by 1.5), and translucent blue points representing outliers. Plot C and D display instability in AUC-ROC values and mean absolute susceptibility prediction error respectively across the 100 runs for each training dataset size – grey points represent each train-test run with horizontal jitter applied for visualisation purposes, and horizontal black lines represent the arithmetic mean value across the 100 runs.

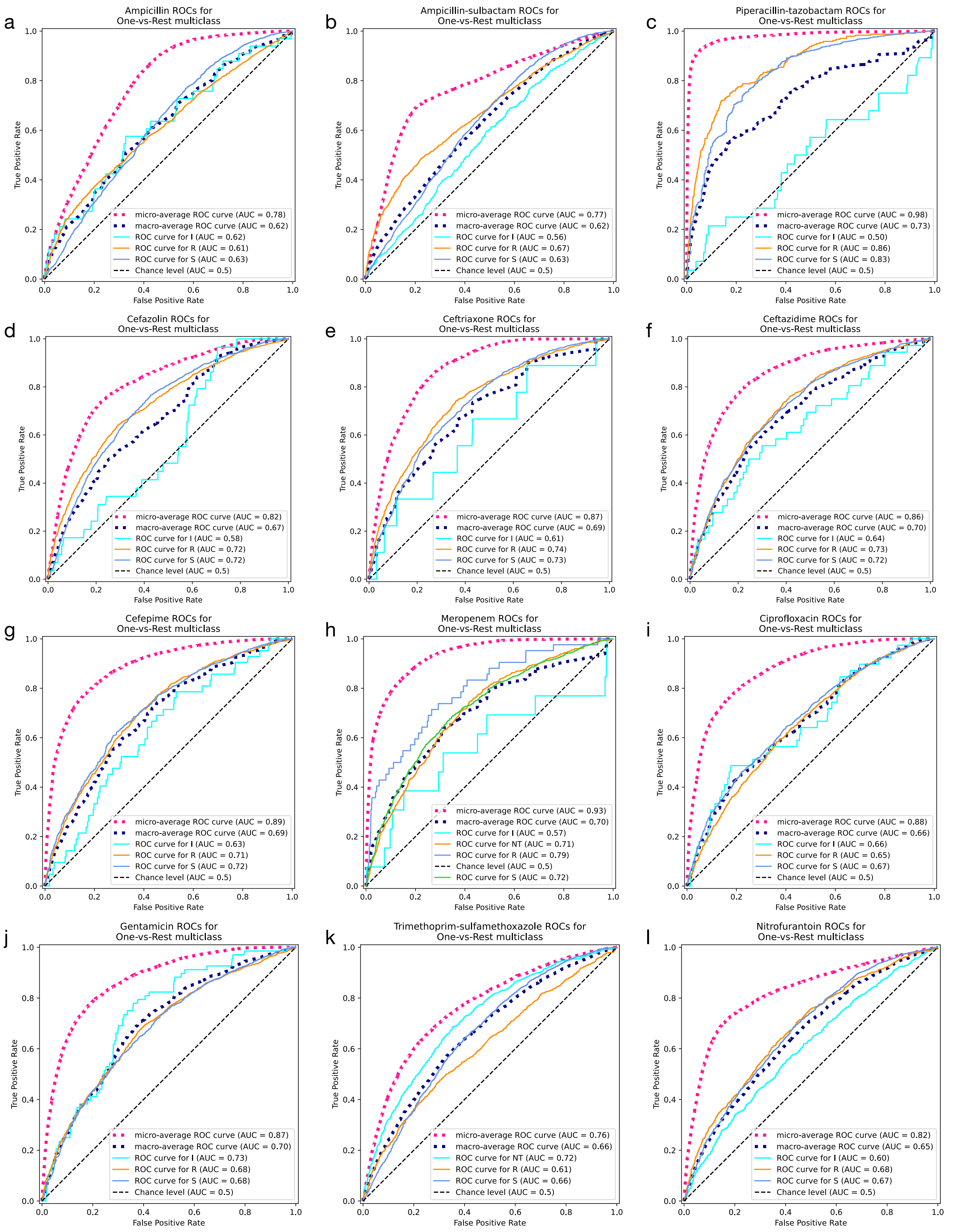

Supplementary Figure 13: Multinomial model performance analysis (n=23,812 patients)**.** Plots A-L correspond to the 12 antimicrobial agents for which susceptibility prediction models were developed. ROC curves and AUC-ROC values are presented for each result class in the data, along with micro-averaged AUC-ROC values (an AUC-ROC value based on all probabilities across classes) and macro-averaged AUC-ROC (arithmetic mean of individual class AUC-ROC values). S = susceptible, I = susceptible at increased exposure, R = resistant, NT = not tested.

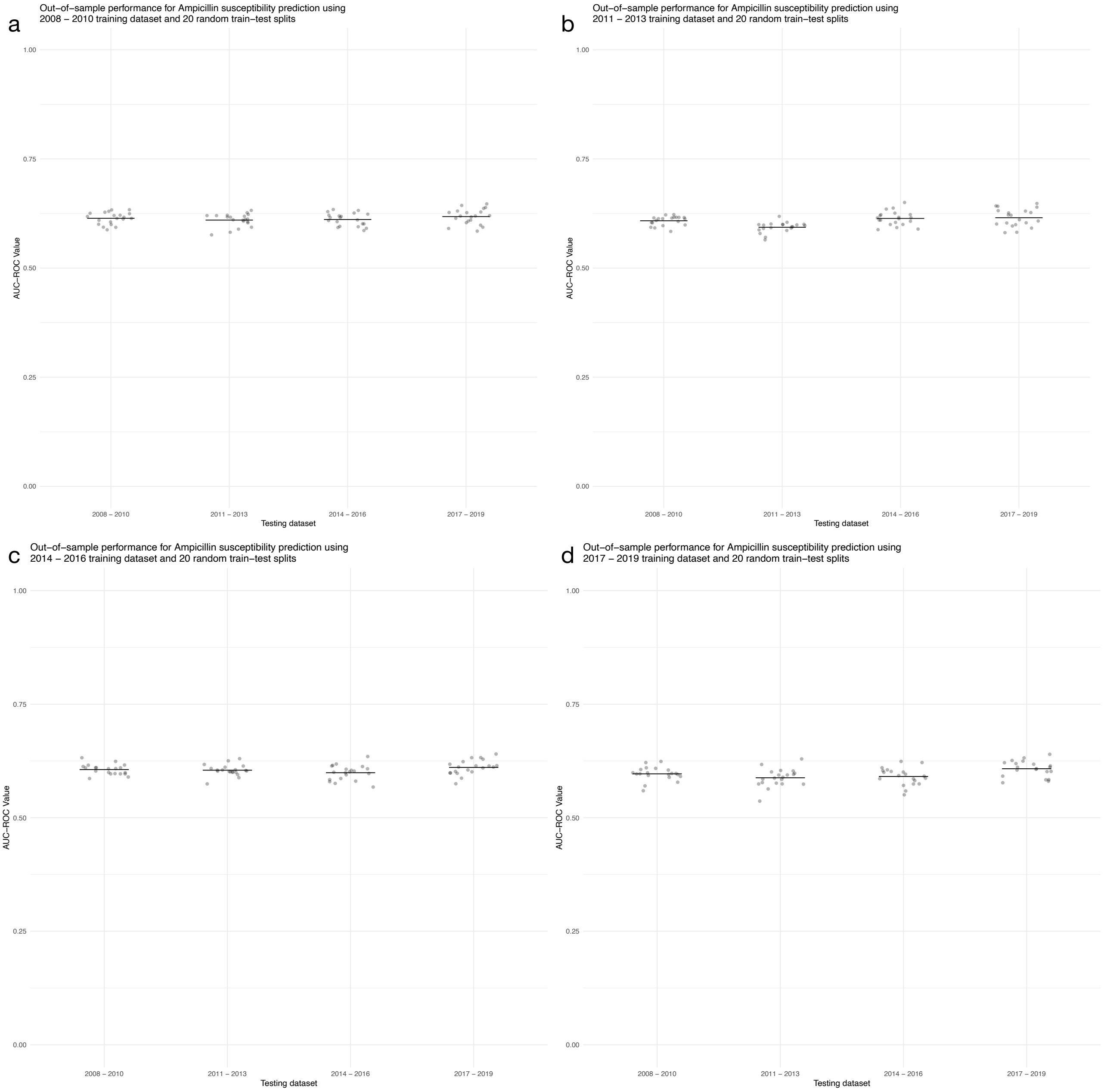

Supplementary Figure 14: Time sensitivity analysis for ampicillin prediction model (n=23,812 patients)**.** Each Plot A-D corresponds to a training dataset from a particular time period. Each dot within the plot corresponds to an AUC-ROC value when a model from that training dataset was validated on the dataset specified on the x-axis, with horizontal jitter applied for visualisation purposes. Each horizontal line corresponds to the arithmetic mean of the 20 random test-train splits done for that particular combination of time periods.

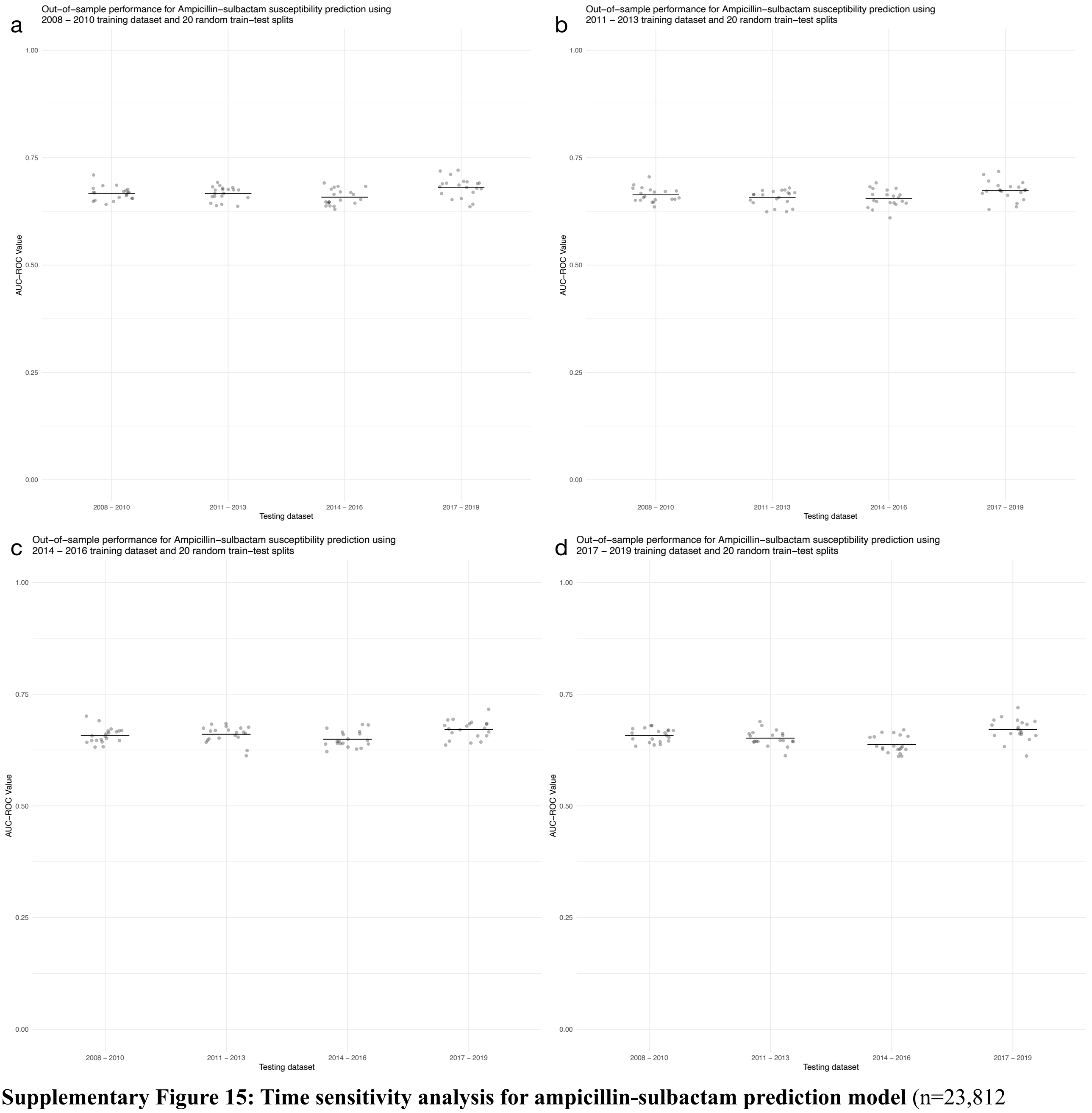

Supplementary Figure 15: Time sensitivity analysis for ampicillin-sulbactam prediction model (n=23,812 patients)**.** Each Plot A-D corresponds to a training dataset from a particular time period. Each dot within the plot corresponds to an AUC-ROC value when a model from that training dataset was validated on the dataset specified on the x-axis, with horizontal jitter applied for visualisation purposes. Each horizontal line corresponds to the arithmetic mean of the 20 random test-train splits done for that particular combination of time periods.

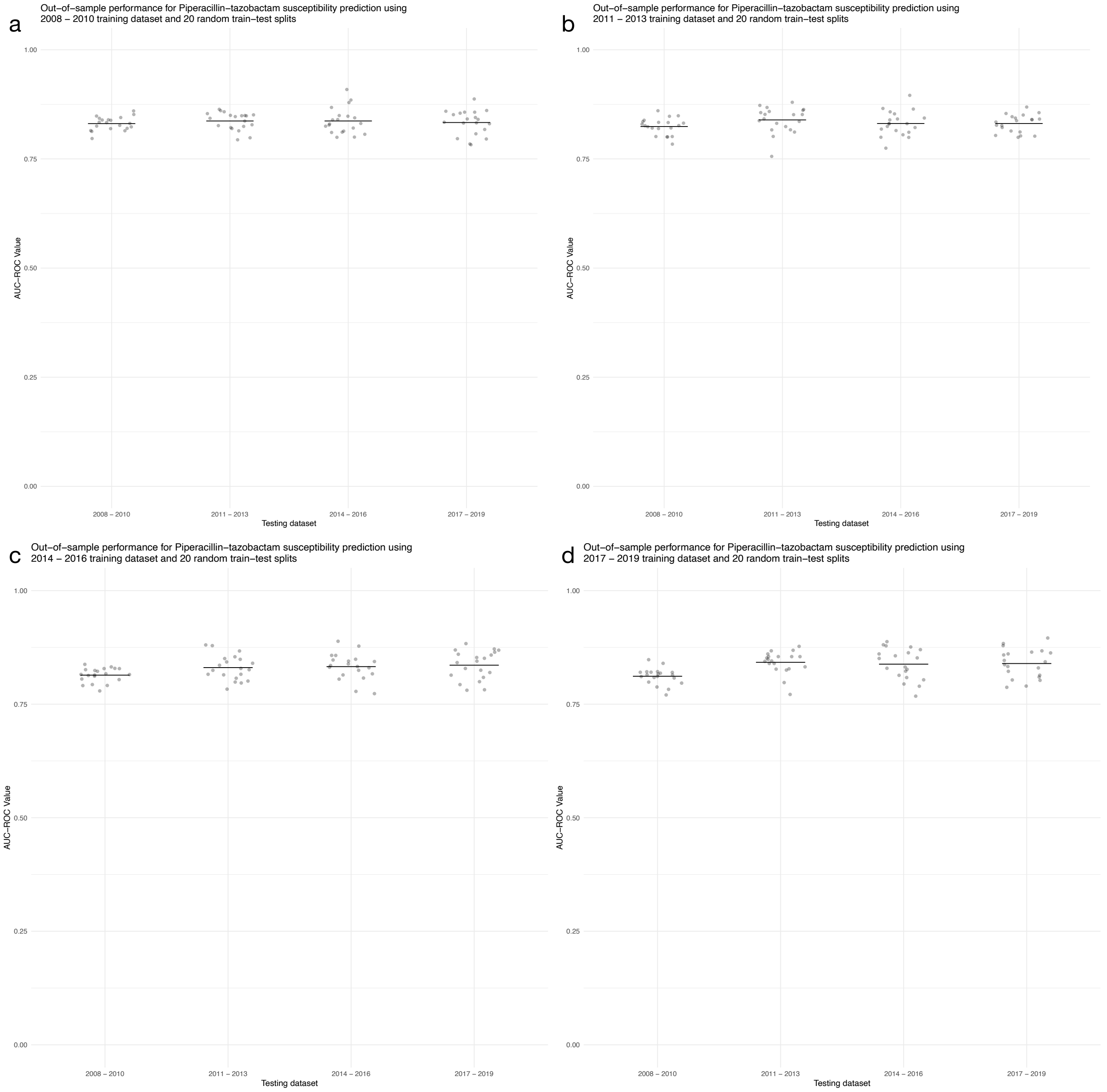

Supplementary Figure 16: Time sensitivity analysis for piperacillin-tazobactam prediction model (n=23,812 patients)**.** Each Plot A-D corresponds to a training dataset from a particular time period. Each dot within the plot corresponds to an AUC-ROC value when a model from that training dataset was validated on the dataset specified on the x-axis, with horizontal jitter applied for visualisation purposes. Each horizontal line corresponds to the arithmetic mean of the 20 random test-train splits done for that particular combination of time periods.

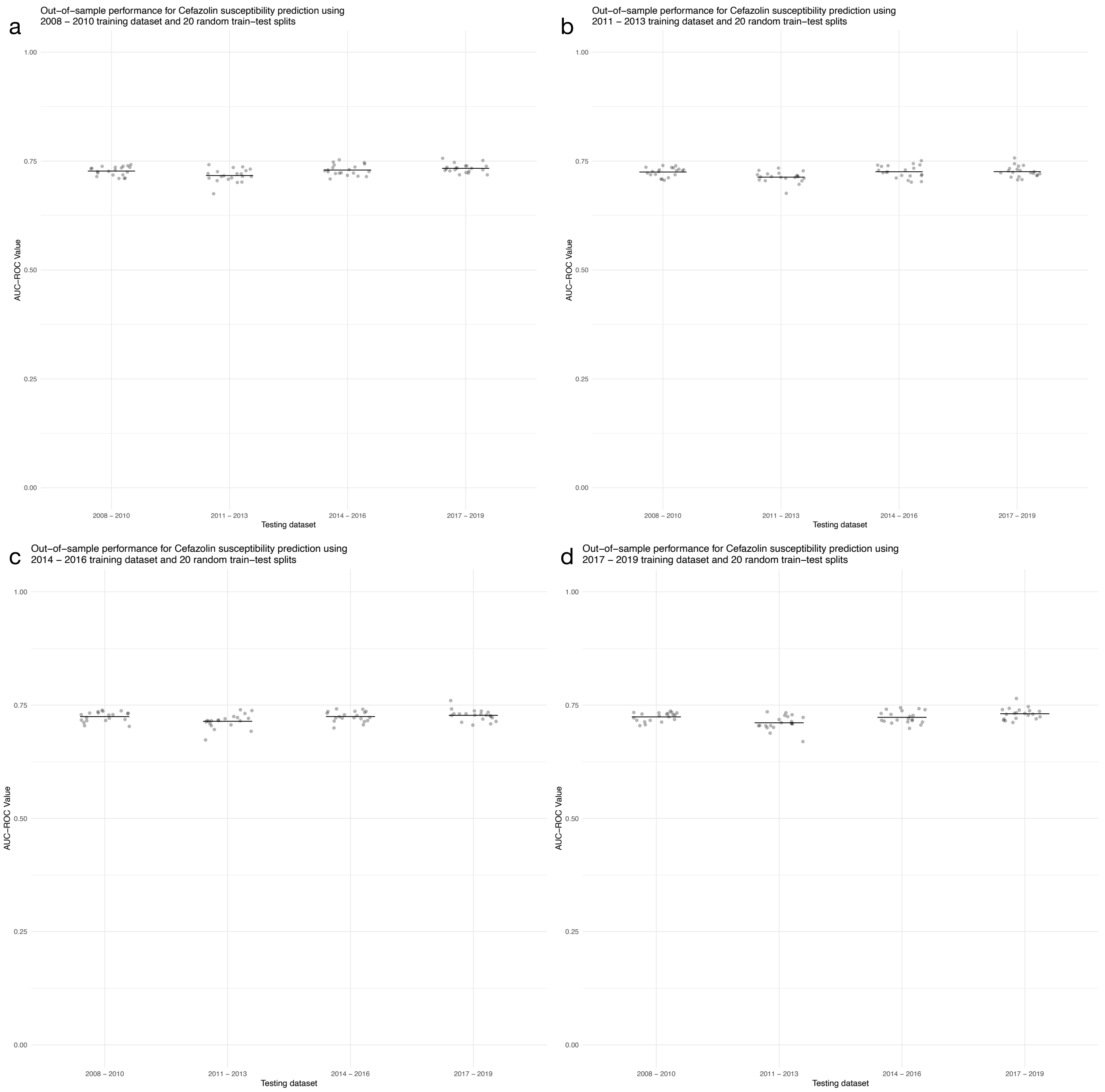

Supplementary Figure 17: Time sensitivity analysis for cefazolin prediction model (n=23,812 patients)**.** Each Plot A-D corresponds to a training dataset from a particular time period. Each dot within the plot corresponds to an AUC-ROC value when a model from that training dataset was validated on the dataset specified on the x-axis, with horizontal jitter applied for visualisation purposes. Each horizontal line corresponds to the arithmetic mean of the 20 random test-train splits done for that particular combination of time periods.

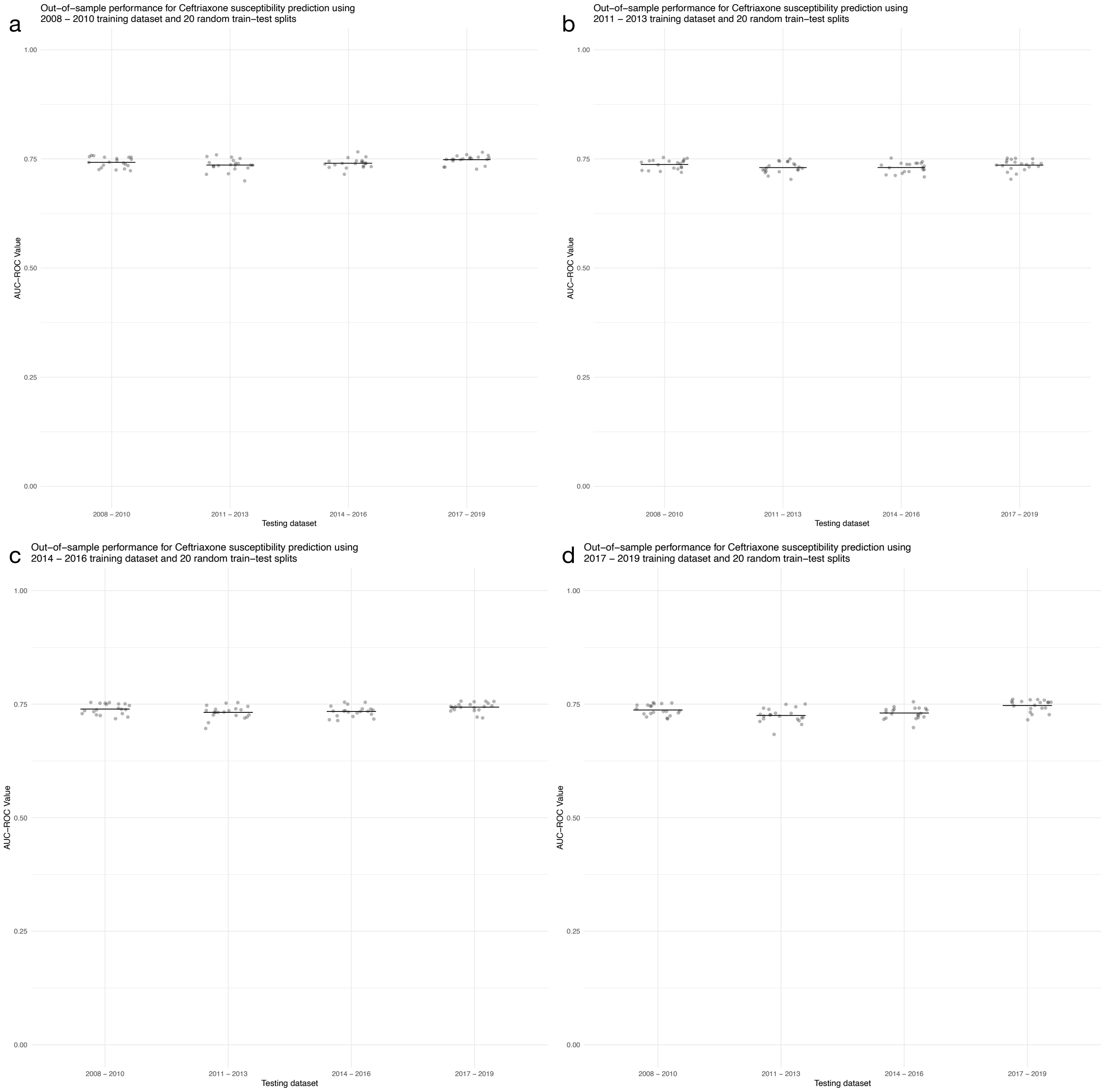

Supplementary Figure 18: Time sensitivity analysis for ceftriaxone prediction model (n=23,812 patients)**.** Each Plot A-D corresponds to a training dataset from a particular time period. Each dot within the plot corresponds to an AUC-ROC value when a model from that training dataset was validated on the dataset specified on the x-axis, with horizontal jitter applied for visualisation purposes. Each horizontal line corresponds to the arithmetic mean of the 20 random test-train splits done for that particular combination of time periods.

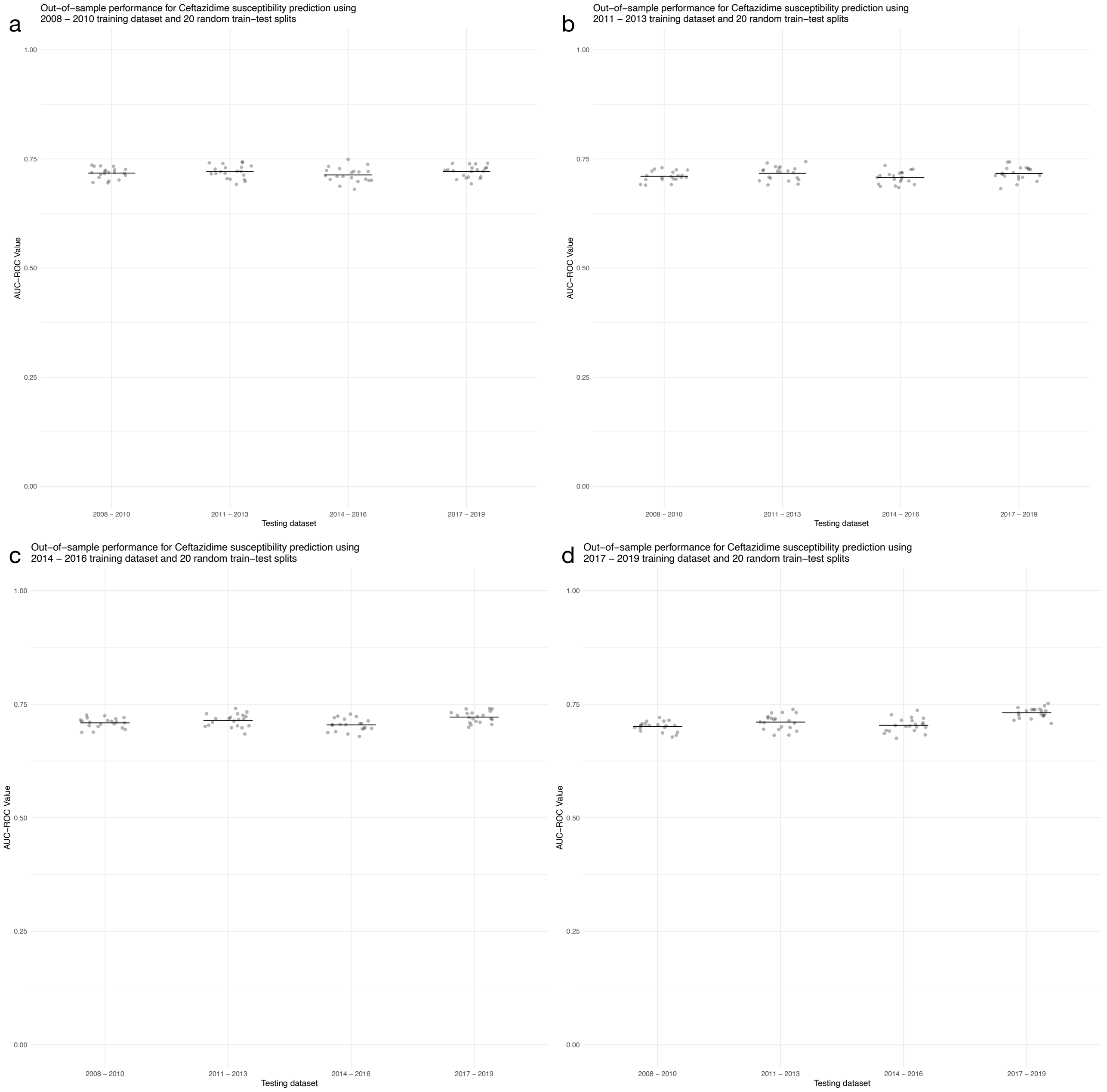

Supplementary Figure 19: Time sensitivity analysis for ceftazidime prediction model (n=23,812 patients)**.** Each Plot A-D corresponds to a training dataset from a particular time period. Each dot within the plot corresponds to an AUC-ROC value when a model from that training dataset was validated on the dataset specified on the x-axis, with horizontal jitter applied for visualisation purposes. Each horizontal line corresponds to the arithmetic mean of the 20 random test-train splits done for that particular combination of time periods.

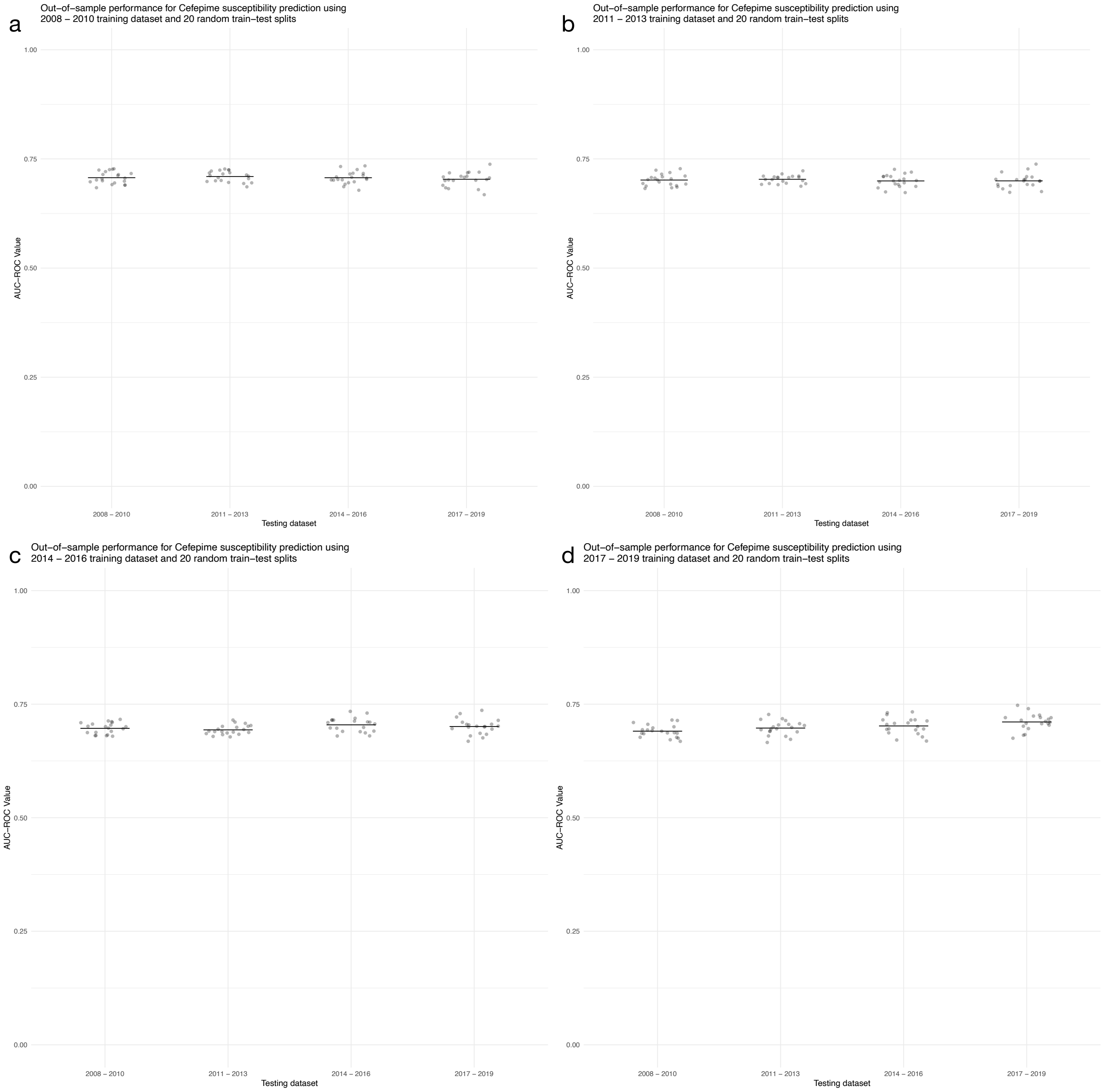

Supplementary Figure 20: Time sensitivity analysis for cefepime prediction model (n=23,812 patients)**.** Each Plot A-D corresponds to a training dataset from a particular time period. Each dot within the plot corresponds to an AUC-ROC value when a model from that training dataset was validated on the dataset specified on the x-axis, with horizontal jitter applied for visualisation purposes. Each horizontal line corresponds to the arithmetic mean of the 20 random test-train splits done for that particular combination of time periods.

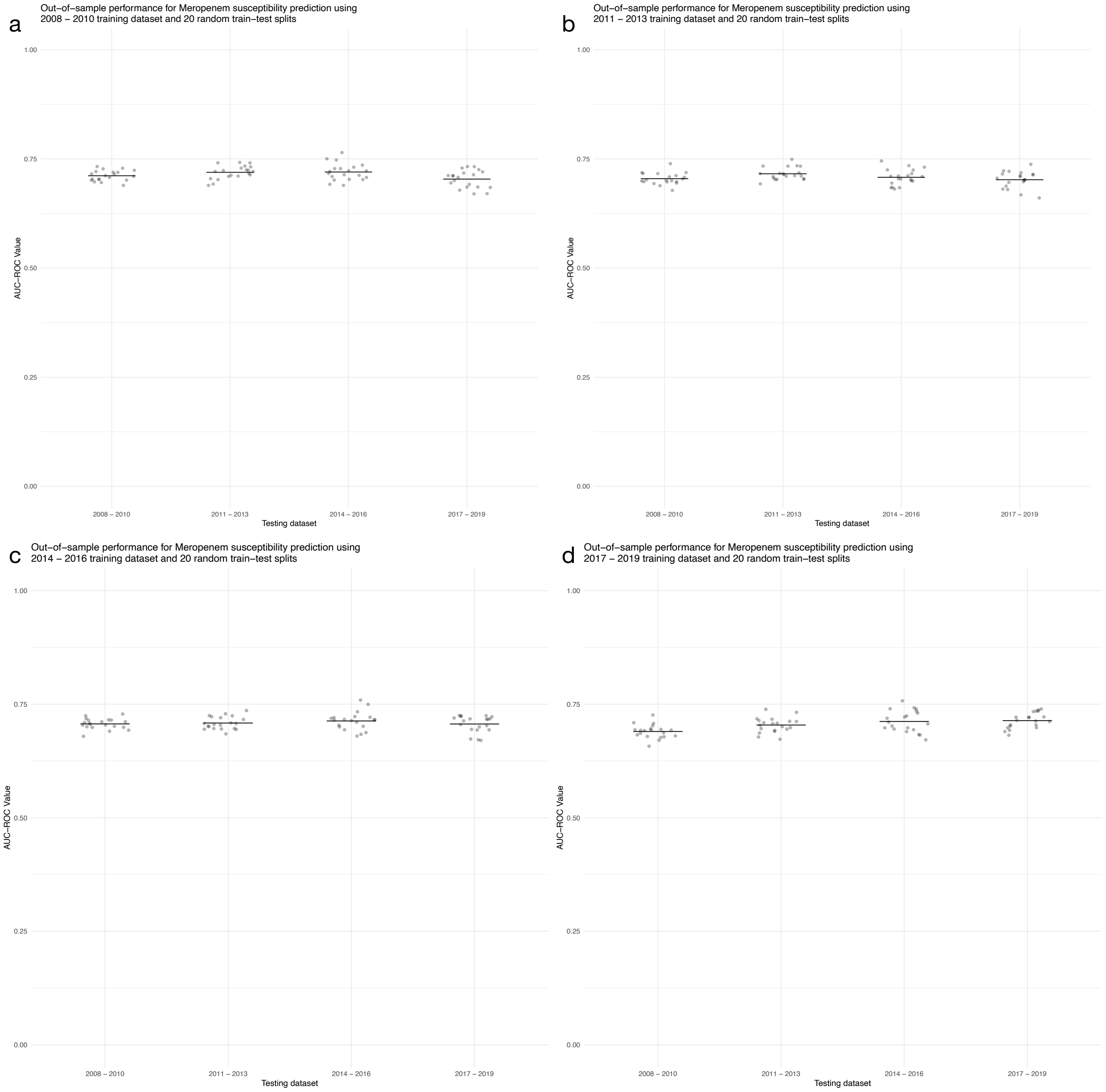

Supplementary Figure 21: Time sensitivity analysis for meropenem prediction model (n=23,812 patients)**.** Each Plot A-D corresponds to a training dataset from a particular time period. Each dot within the plot corresponds to an AUC-ROC value when a model from that training dataset was validated on the dataset specified on the x-axis, with horizontal jitter applied for visualisation purposes. Each horizontal line corresponds to the arithmetic mean of the 20 random test-train splits done for that particular combination of time periods.

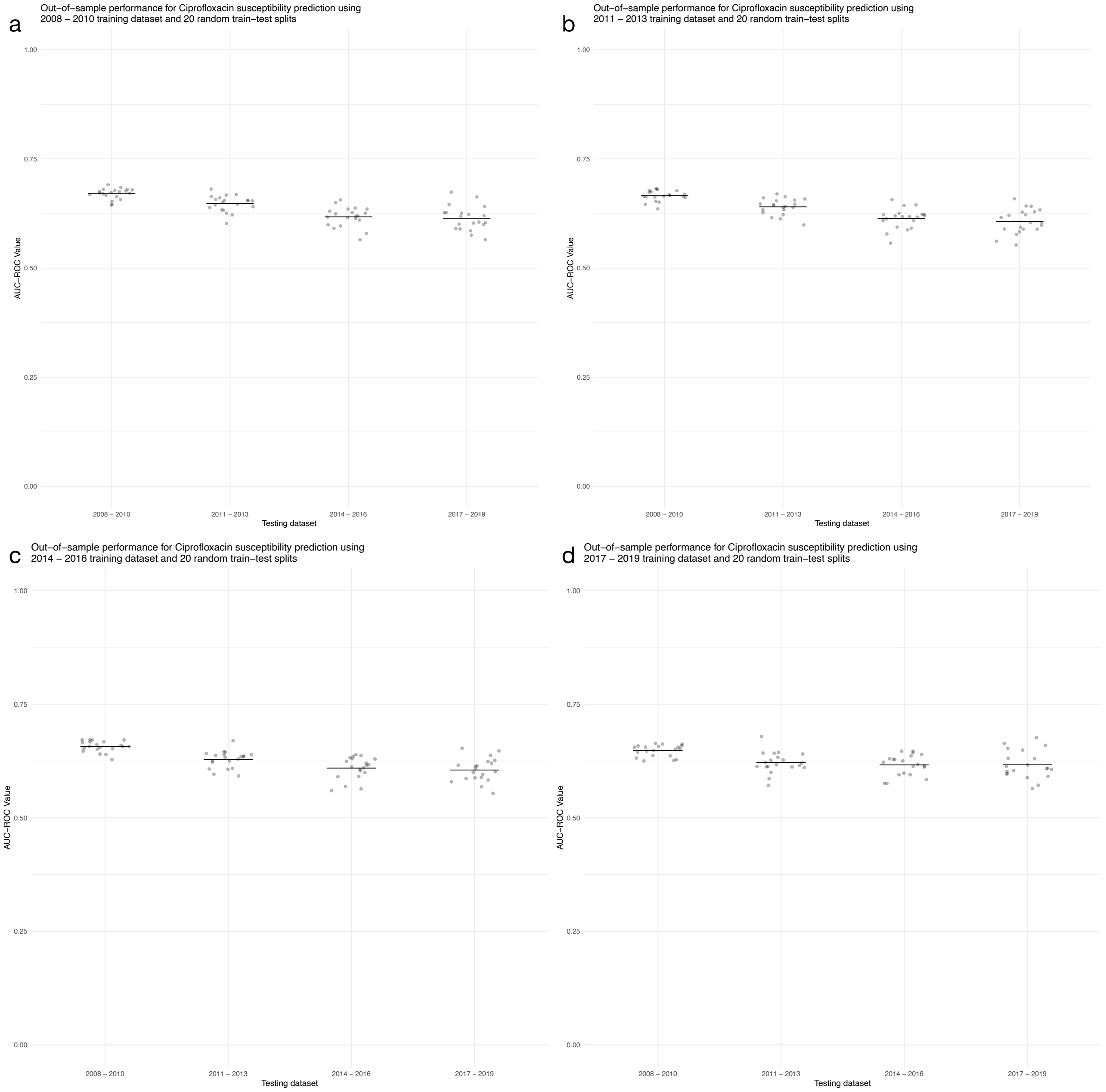

Supplementary Figure 22: Time sensitivity analysis for ciprofloxacin prediction model (n=23,812 patients)**.** Each Plot A-D corresponds to a training dataset from a particular time period. Each dot within the plot corresponds to an AUC-ROC value when a model from that training dataset was validated on the dataset specified on the x-axis, with horizontal jitter applied for visualisation purposes. Each horizontal line corresponds to the arithmetic mean of the 20 random test-train splits done for that particular combination of time periods.

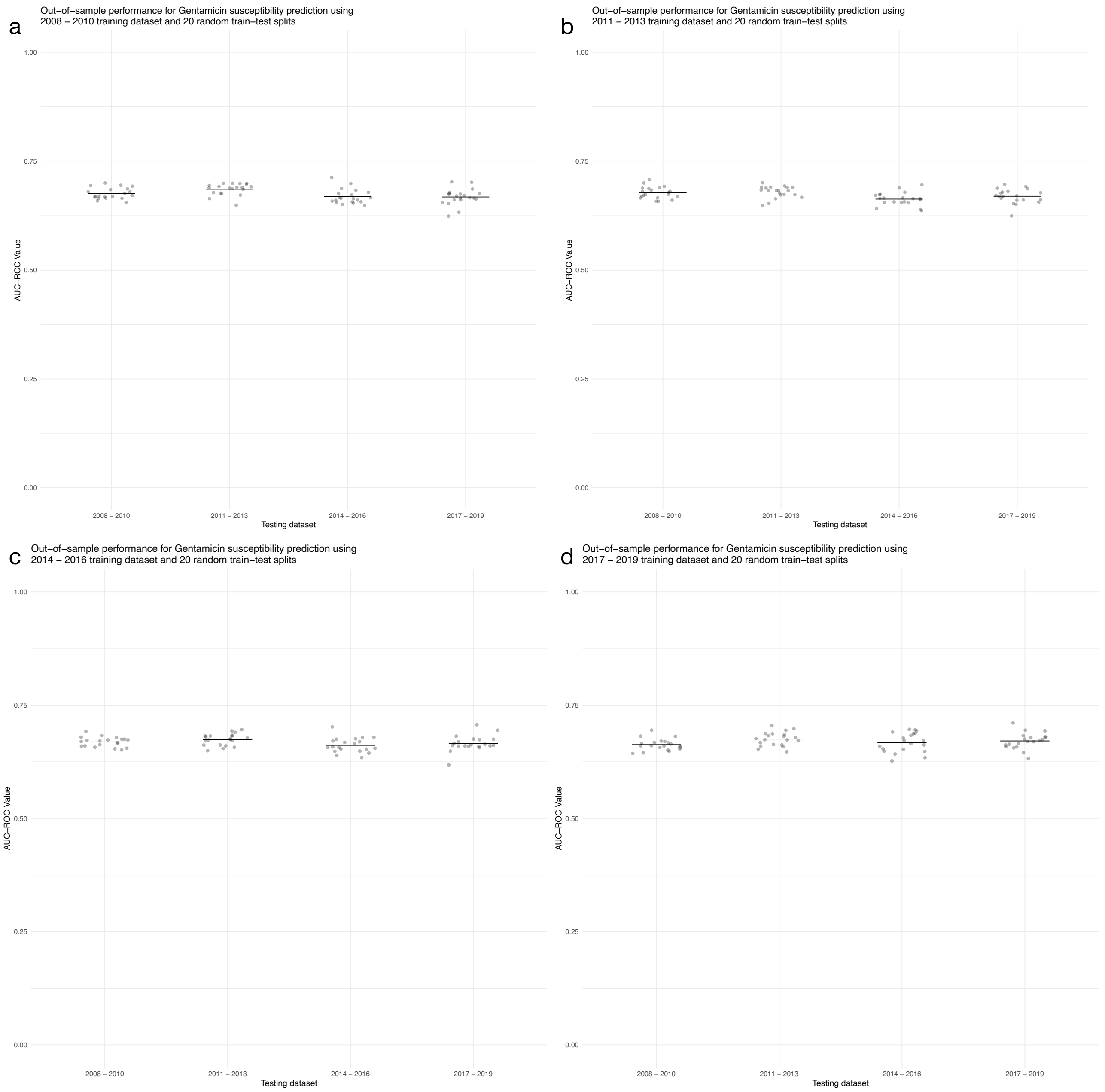

Supplementary Figure 23: Time sensitivity analysis for gentamicin prediction model (n=23,812 patients)**.** Each Plot A-D corresponds to a training dataset from a particular time period. Each dot within the plot corresponds to an AUC-ROC value when a model from that training dataset was validated on the dataset specified on the x-axis, with horizontal jitter applied for visualisation purposes. Each horizontal line corresponds to the arithmetic mean of the 20 random test-train splits done for that particular combination of time periods.

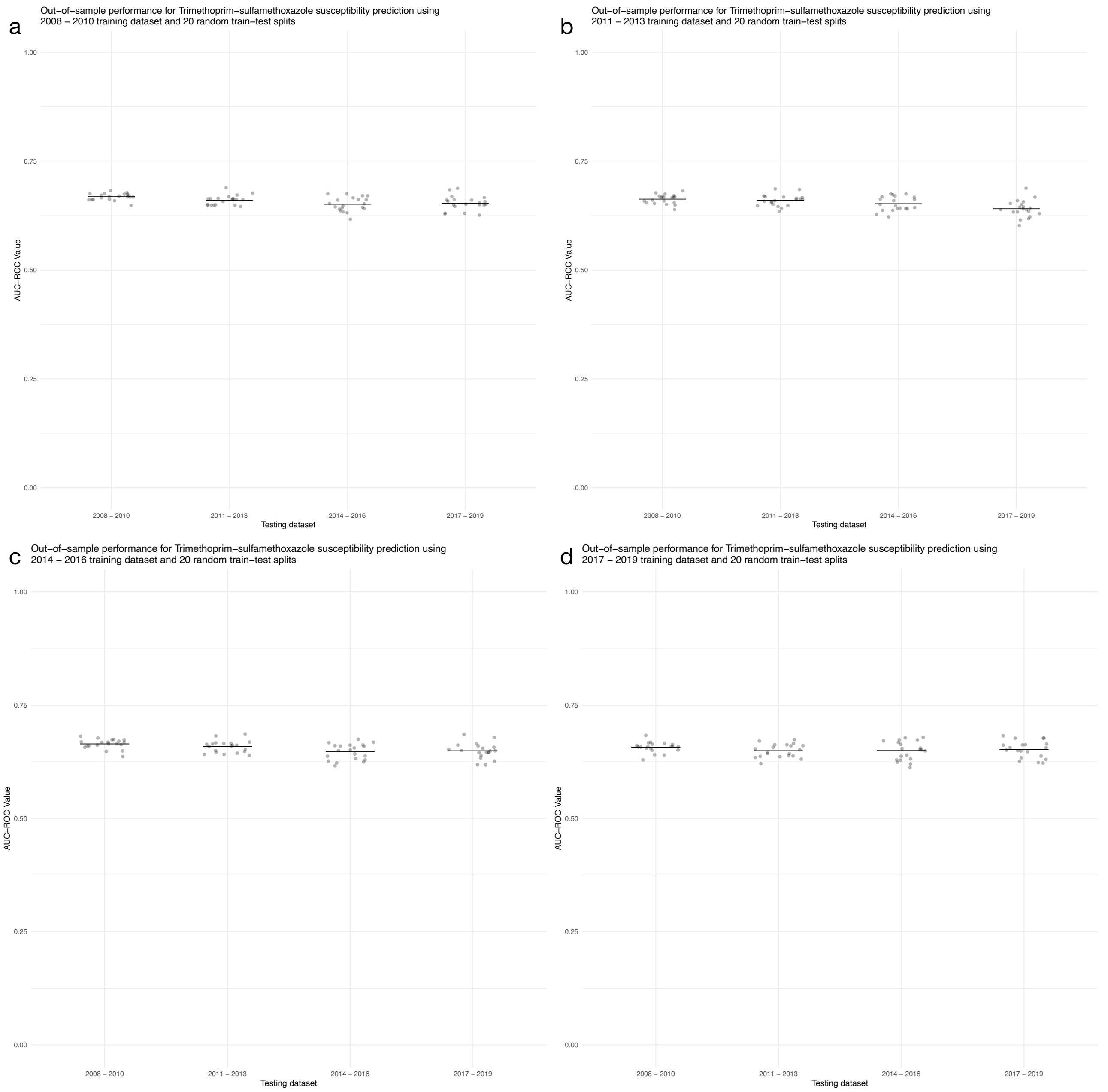

Supplementary Figure 24: Time sensitivity analysis for trimethoprim-sulfamethoxazole prediction model (n=23,812 patients)**.** Each Plot A-D corresponds to a training dataset from a particular time period. Each dot within the plot corresponds to an AUC-ROC value when a model from that training dataset was validated on the dataset specified on the x-axis, with horizontal jitter applied for visualisation purposes. Each horizontal line corresponds to the arithmetic mean of the 20 random test-train splits done for that particular combination of time periods.

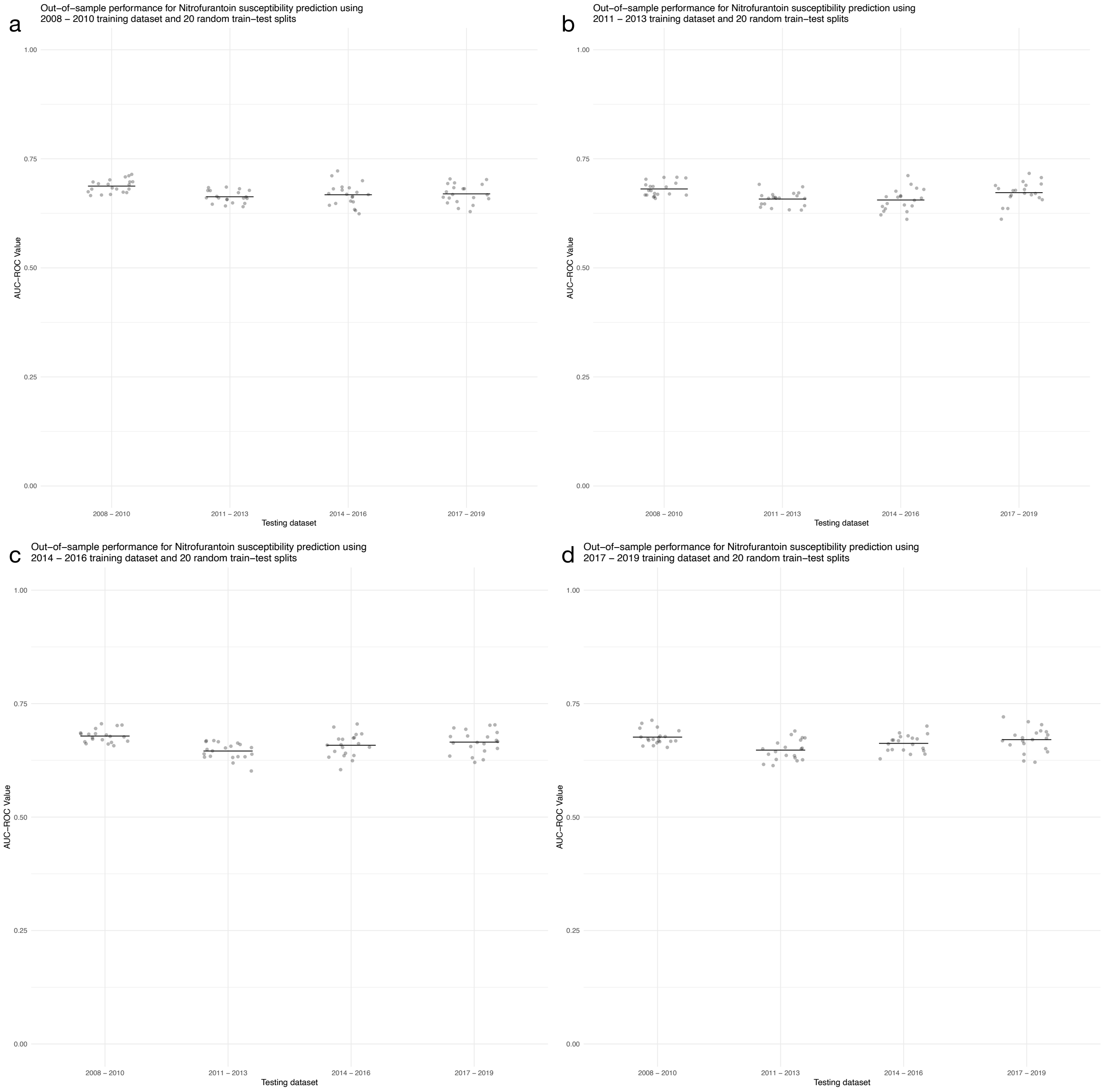

Supplementary Figure 25: Time sensitivity analysis for nitrofurantoin prediction model (n=23,812 patients)**.** Each Plot A-D corresponds to a training dataset from a particular time period. Each dot within the plot corresponds to an AUC-ROC value when a model from that training dataset was validated on the dataset specified on the x-axis, with horizontal jitter applied for visualisation purposes. Each horizontal line corresponds to the arithmetic mean of the 20 random test-train splits done for that particular combination of time periods.

a

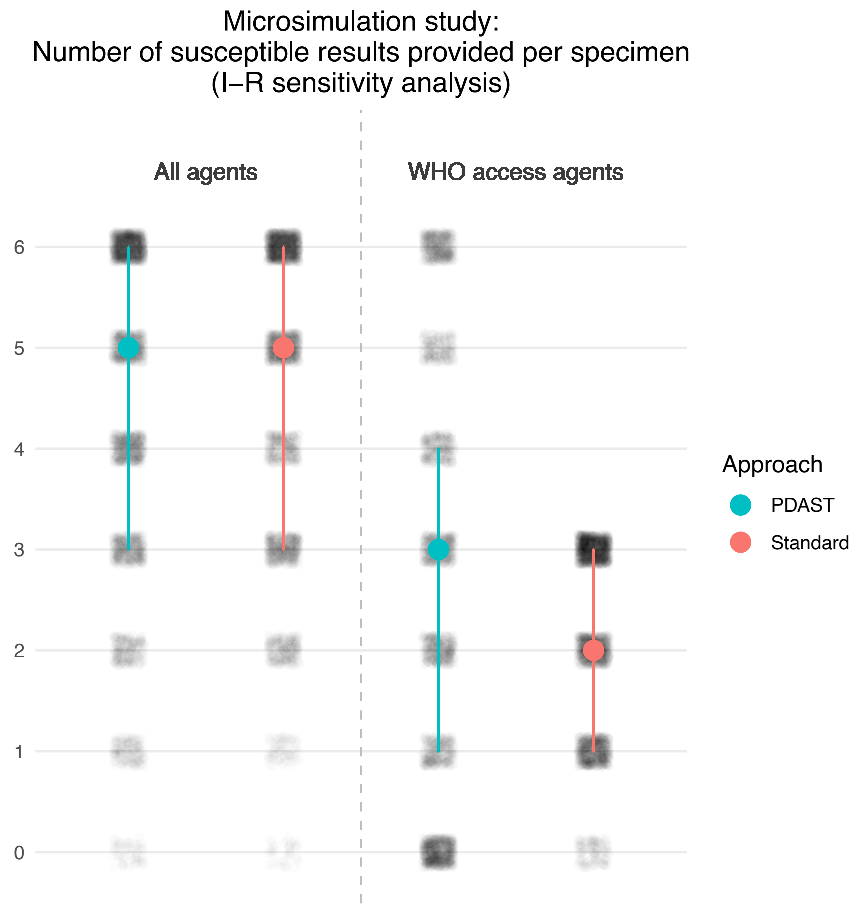

b

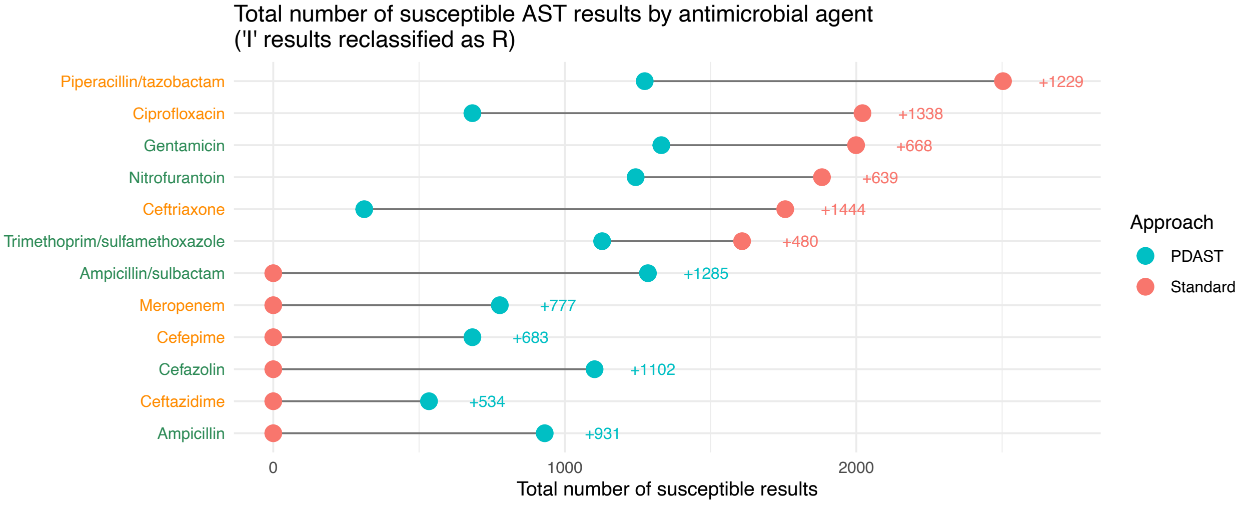

Supplementary Figure 26: Microsimulation sensitivity analysis where all 'I' results were reclassified as R (main sub-analysis) (n=2,646 patients)**.** Plot A displays the number of all susceptible results and susceptible results for WHO Access agents provided by the personalised and standard approaches. Plot B displays the number of susceptible results provided for each antimicrobial agent by the personalised and standard approaches.

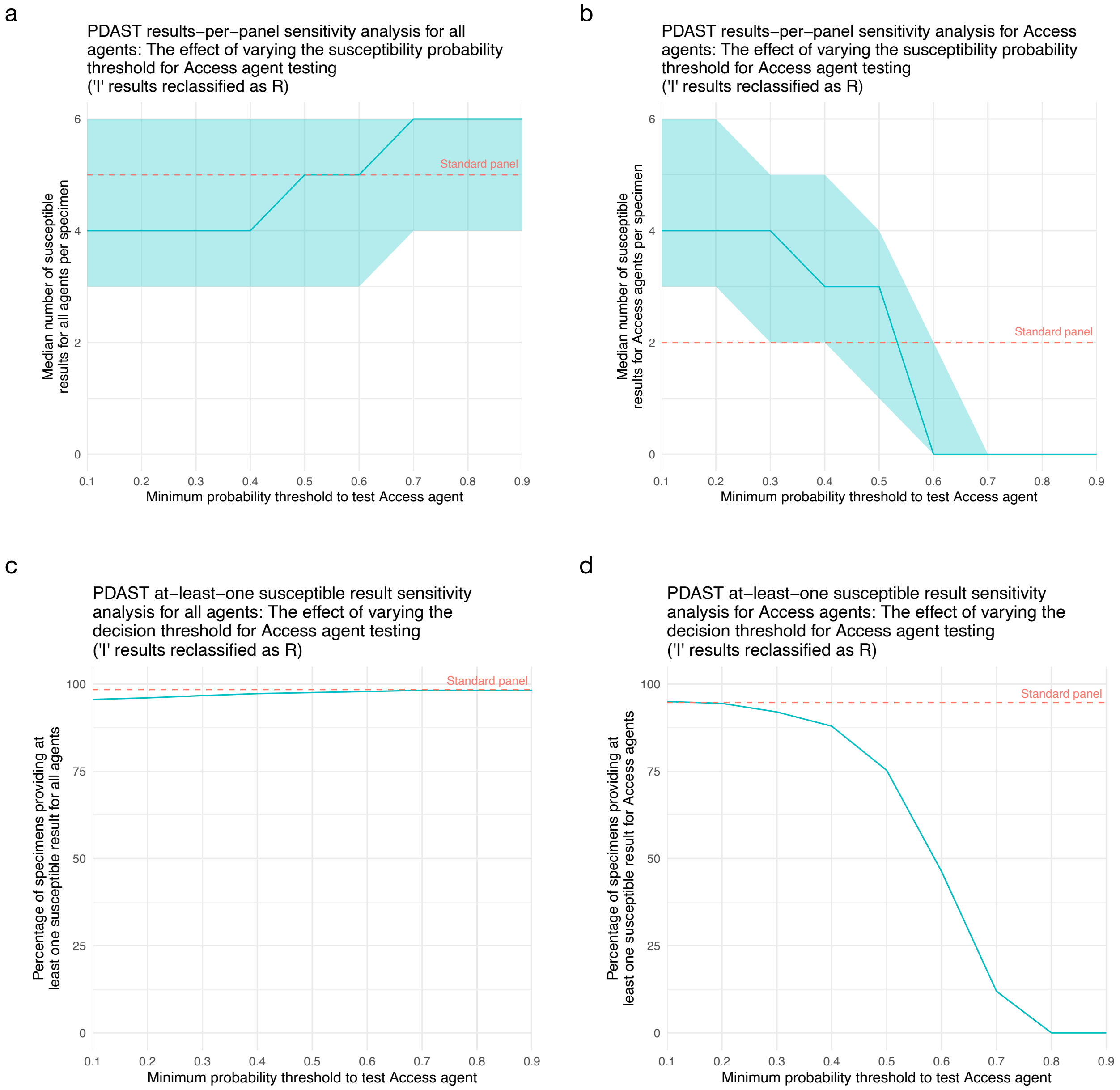

Supplementary Figure 27: Microsimulation sensitivity analysis where all 'I' results were reclassified as R (decision threshold sub-analysis) (n=2,646 patients)**.** Plots A and B display the effect of varying the testing decision threshold on the number of susceptible results provided per panel for all agents and WHO Access category agents respectively. Plots c and d display the effect of varying the testing decision threshold on the percentage of panels that provide at least one susceptible result for all agents and WHO Access category agents respectively. In plots c and d, blue lines represent the median number of susceptible results per panel for the personalised approach (PDAST) and shaded areas represent the interquartile range. In plots C and D, blue lines represent the percentage of specimens providing at least one susceptible result. Red dashed lines represent the median number of susceptible results per specimen (top two graphs) and the percentage of specimens with at least one susceptible result (bottom two graphs) provided by the standard comparator panel.

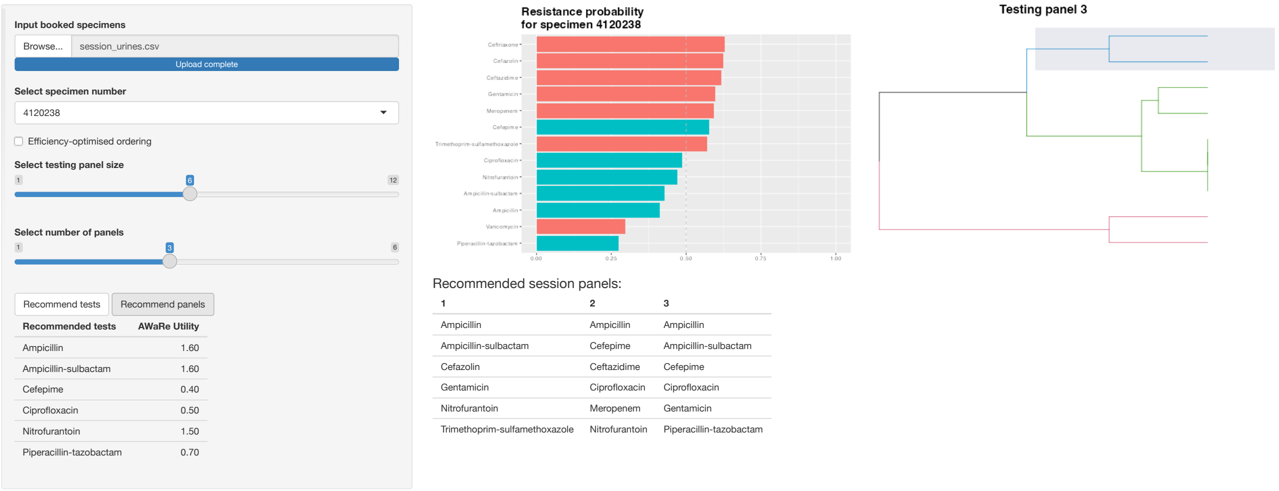

Supplementary Figure 28: ADAPT-AST application prototype. A screenshot of an RShiny (https://cran.r-project.org/web/packages/shiny/index.html) prototype user interface developed by the authors that shows how a personalised approach could be used by the microbiology laboratory to individualise antimicrobial susceptibility testing.[8]

Probabilities of antimicrobial susceptibility are calculated based on individual patients' previous electronic healthcare record data using statistical clinical prediction methods – these probabilities are then used to prioritise antimicrobials for inclusion in a chosen testing panel size based on their utility, which here is defined as their ability to provide susceptible results, with WHO Access agents prioritised for selection if they meet a susceptibility probability decision threshold. Key metrics are also be provided to aid with interpretation in the clinical context (here, a bar chart of probability of resistance, with agents recommended for testing highlighted in green).
