# Supplementary material for: Personalised antimicrobial susceptibility testing with clinical prediction modelling informs appropriate antibiotic use
Source: Nat Commun. 2024 Nov 21;15:9924. doi: 10.1038/s41467-024-54192-3 (PMC11582675; doi:10.1038/s41467-024-54192-3)
Supplement: Supplementary file 3 — Description of Additional Supplementary Files [file 41467_2024_54192_MOESM3_ESM.pdf]

Description of Additional Supplementary Files

Supplementary Data 1: Characteristics of the study population. IQR = interquartile range, S = susceptible, I = susceptible at increased exposure/intermediate, R = resistant, NT = not testable. *MIMIC-IV provides a column listed ‘gender ’with the binary elements M ’and ‘F’. In the absence of further available information, we have assumed they refer to the terms male and female, but we have not assumed whether this refers to gender or sex and have summarised the data as provided.

Supplementary Data 2: Datasets, variables, and associated coefficients for the main analysis. The top section details the CSV files from the PhysioNet MIMIC-IV 2.2 database used to derive predictor feature variables. The subsequent 12 table sections summarise the model parameters and coefficients for susceptibility prediction in the final binary logistic regression models validated in the main analysis and used to perform the microsimulation study. In each section, the coefficient is presented first, then coefficients are listed in descending order. Positive coefficients suggest association of the corresponding feature variable with an increased probability of susceptibility to that antimicrobial agents, and negative coefficients suggest association with an increase probability of resistance.

Supplementary Data 3: Model fairness analysis. Performance characteristics of the trained model for a single validation run in patients with specific demographic characteristics. n = number of subjects with that characteristic in the model development dataset, TPR = true positive rate, FPR = false positive rate, FNR = false negative rate, PPR = predicted-as-positive rate
