# Supplementary material for: Personalised antimicrobial susceptibility testing with clinical prediction modelling informs appropriate antibiotic use
Source: Nat Commun. 2024 Nov 21;15:9924. doi: 10.1038/s41467-024-54192-3 (PMC11582675; doi:10.1038/s41467-024-54192-3)
Supplement: Supplementary file 7 — Reporting Summary [file 41467_2024_54192_MOESM7_ESM.pdf]

nature portfolio

Corresponding author(s): Alex Howard

Last updated by author(s): Jul 30, 2024

Reporting Summary

Nature Portfolio wishes to improve the reproducibility of the work that we publish. This form provides structure for consistency and transparency in reporting. For further information on Nature Portfolio policies, see our Editorial Policies and the Editorial Policy Checklist.

Statistics

For all statistical analyses, confirm that the following items are present in the figure legend, table legend, main text, or Methods section.

n/a	Confirmed	
☐	☒	The exact sample size (*n*) for each experimental group/condition, given as a discrete number and unit of measurement
☐	☒	A statement on whether measurements were taken from distinct samples or whether the same sample was measured repeatedly
☐	☒	The statistical test(s) used AND whether they are one- or two-sided<br>*Only common tests should be described solely by name; describe more complex techniques in the Methods section.*
☐	☒	A description of all covariates tested
☐	☒	A description of any assumptions or corrections, such as tests of normality and adjustment for multiple comparisons
☐	☒	A full description of the statistical parameters including central tendency (e.g. means) or other basic estimates (e.g. regression coefficient) AND variation (e.g. standard deviation) or associated estimates of uncertainty (e.g. confidence intervals)
☐	☒	For null hypothesis testing, the test statistic (e.g. *F*, *t*, *r*) with confidence intervals, effect sizes, degrees of freedom and *P* value noted<br>*Give P values as exact values whenever suitable.*
☒	☐	For Bayesian analysis, information on the choice of priors and Markov chain Monte Carlo settings
☐	☒	For hierarchical and complex designs, identification of the appropriate level for tests and full reporting of outcomes
☐	☒	Estimates of effect sizes (e.g. Cohen's *d*, Pearson's *r*), indicating how they were calculated

Our web collection on statistics for biologists contains articles on many of the points above.

Software and code

Policy information about availability of computer code

Data collection	The open-source data used were collected from PhysioNet (https://physionet.org/content/mimiciv/2.2/) according to the ethical and other terms of the License and Data Use Agreement by direct download from the URL.
Data analysis	Custom code was written and run using R version 4.3.2 and Python version 3.12.0.<br>Full code is available at https://github.com/amh312/PDAST, with full instructions on running of the code included in the README file.

For manuscripts utilizing custom algorithms or software that are central to the research but not yet described in published literature, software must be made available to editors and reviewers. We strongly encourage code deposition in a community repository (e.g. GitHub). See the Nature Portfolio guidelines for submitting code & software for further information.

Data

Policy information about availability of data

All manuscripts must include a data availability statement. This statement should provide the following information, where applicable:

- Accession codes, unique identifiers, or web links for publicly available datasets
- A description of any restrictions on data availability
- For clinical datasets or third party data, please ensure that the statement adheres to our policy

The data are publicly available from https://physionet.org/content/mimiciv/2.2/ subject to the completion of credentialing tasks.

Research involving human participants, their data, or biological material

Policy information about studies with human participants or human data. See also policy information about sex, gender (identity/presentation), and sexual orientation and race, ethnicity and racism.

Reporting on sex and gender	MIMIC-IV provides a column listed 'gender' with the binary elements 'M' and 'F'. In the absence of further available information, we have not assumed whether this refers to gender or sex and have summarised the data as provided. A full fairness analysis was performed that assessed model performance on all the different demographic categories present in the data.
Reporting on race, ethnicity, or other socially relevant groupings	MIMIC-IV provides a column listed 'race' - we have summarised the data as provided. A full fairness analysis was performed that assessed model performance on all the different demographic categories present in the data.
Population characteristics	MIMIC-IV provides a column listed 'age' - we have summarised the data as provided. A full fairness analysis was performed that assessed model performance on all the different demographic categories present in the data.
Recruitment	Patients were selected from the MIMIC-IV dataset based on the presence of positive urine culture results. The dataset pertains to inpatients and outpatients who had any emergency department and/or intensive care admissions over the time period of the study dataset. There may therefore be an element of selection bias in that the rates of antimicrobial resistance may be higher than the general population - however, it is this cohort of patients in which the personalised AST approach would be likely to have the most clinical benefit.
Ethics oversight	The data were accessed and used according to the ethical regulations stipluated in the License and Data Use Agreement for the publicly available MIMIC-IV dataset used.

Note that full information on the approval of the study protocol must also be provided in the manuscript.

Field-specific reporting

Please select the one below that is the best fit for your research. If you are not sure, read the appropriate sections before making your selection.

☒ Life sciences ☐ Behavioural & social sciences ☐ Ecological, evolutionary & environmental sciences

For a reference copy of the document with all sections, see nature.com/documents/nr-reporting-summary-flat.pdf

Life sciences study design

All studies must disclose on these points even when the disclosure is negative.

Sample size	Sample size was determined by the number of rows present in the dataset once filtered to positive urine results, then filtered to the last urine result for each patient.
Data exclusions	Rows of data that did not pertain to positive urine culture results were not used in the analysis.
Replication	Model and microsimulation study sensitivity analyses were performed in the form of a small dataset stability analysis, a model fairness analysis, an analysis where all 'I' results were reclassified as 'R', a specimen pathway information availability analysis, and a multinomial model analysis - all of these analyses required repeated running and testing of the model.
Randomization	Randomisation without replacement was performed to split data into a training/testing dataset, a model validation dataset, and a microsimulation study dataset. Randomisation of the model development dataset was stratified to ensure similar outcome proportions in the training, testing, and validation datasets.
Blinding	Blinding was not relevant to the study because the microsimulation study design meant both the standard and personalised approaches could be applied to the same rows of data.

Reporting for specific materials, systems and methods

We require information from authors about some types of materials, experimental systems and methods used in many studies. Here, indicate whether each material, system or method listed is relevant to your study. If you are not sure if a list item applies to your research, read the appropriate section before selecting a response.

Materials & experimental systems

n/a	Involved in the study
☒	☐ Antibodies
☒	☐ Eukaryotic cell lines
☒	☐ Palaeontology and archaeology
☒	☐ Animals and other organisms
☒	☐ Clinical data
☒	☐ Dual use research of concern
☒	☐ Plants

Methods

n/a	Involved in the study
☒	☐ ChIP-seq
☒	☐ Flow cytometry
☒	☐ MRI-based neuroimaging

Plants

Seed stocks

Report on the source of all seed stocks or other plant material used. If applicable, state the seed stock centre and catalogue number. If plant specimens were collected from the field, describe the collection location, date and sampling procedures.

Novel plant genotypes

Describe the methods by which all novel plant genotypes were produced. This includes those generated by transgenic approaches, gene editing, chemical/radiation-based mutagenesis and hybridization. For transgenic lines, describe the transformation method, the number of independent lines analyzed and the generation upon which experiments were performed. For gene-edited lines, describe the editor used, the endogenous sequence targeted for editing, the targeting guide RNA sequence (if applicable) and how the editor was applied.

Authentication

Describe any authentication procedures for each seed stock used or novel genotype generated. Describe any experiments used to assess the effect of a mutation and, where applicable, how potential secondary effects (e.g. second site T-DNA insertions, mosiacism, off-target gene editing) were examined.
